# Shrimp Virus Regulates ROS Dynamics via the Nrf2 Pathway to Facilitate Viral Replication

**DOI:** 10.1002/advs.202407695

**Published:** 2025-03-16

**Authors:** Honghui He, Kai Yuan, Junming Pan, Shaoping Weng, Chaozheng Li, Yihong Chen, Jianguo He

**Affiliations:** ^1^ State Key Laboratory of Biocontrol School of Marine Sciences Sun Yat‐sen University Guangzhou Guangdong 510275 China; ^2^ School of Life Sciences Sun Yat‐sen University Guangzhou Guangdong 510275 China; ^3^ China‐ASEAN Belt and Road Joint Laboratory on Mariculture Technology Southern Marine Sciences and Engineering Guangdong Laboratory (Zhuhai) Zhuhai Guangdong 519000 China; ^4^ School of Life Sciences Huizhou University Huizhou Guangdong 516007 China; ^5^ Institute of Modern Aquaculture Science and Engineering College of Life Sciences South China Normal University Guangzhou Guangdong 510631 China

**Keywords:** rf2 pathway, reactive oxygen species, redox homeostasis, shrimp, white spot syndrome virus

## Abstract

Reactive oxygen species (ROS) of hosts are widely involved in intracellular signaling and against pathogens. Viruses manipulate ROS homeostasis of hosts as a strategy to evade ROS‐mediated negative effects of their infection, but the mechanisms remain unclear. The economically important aquaculture shrimp, *Litopenaeus vannamei*, is selected to investigate the molecular mechanism of how white spot syndrome virus (WSSV) regulates ROS dynamics and enhances viral replication. WSSV protein wsv220 binds to the repressor of shrimp nuclear factor erythroid 2‐related factor 2 (LvNrf2), called Kelch‐like ECH‐associated protein 1 (LvKeap1), disrupting LvNrf2/LvKeap1 complex and facilitating LvNrf2 nuclear translocation. This activation of LvNrf2 causes up‐regulation of antioxidant genes, including glucose‐6‐phosphate dehydrogenase (LvG6PDH), which increases nicotinamide adenine dinucleotide phosphate (NADPH) and glutathione (GSH) production, effectively eliminating excessive ROS. Moreover, WSSV exploits LvNrf2 to establish a positive feedback loop by up‐regulating viral immediate early gene wsv051, which further enhances wsv220 expression. Knockdown of LvNrf2 or LvG6PDH reduces WSSV replication and increases host ROS levels. Therefore, WSSV hijacks LvNrf2 pathway to maintain ROS homeostasis and establishes a positive feedback loop to facilitate WSSV replication. These findings reveal a novel molecular mechanism of viral manipulation of host ROS dynamics and suggest potential antiviral strategies targeting LvNrf2 pathway.

## Graphical abstract

Viruses manipulate host ROS as a strategy to achieve successful infection. WSSV exploits Nrf2 pathway to maintain ROS homeostasis and establishes a positive feedback loop to facilitate WSSV replication. Wsv220 competitively binds to Keap1 to activate Nrf2. Nrf2 up‐regulates LvG6PDH to produce more NADPH and GSH to eliminate excessive ROS. The feedback loop of wsv220‐Nrf2‐wsv051 favors rapid viral replication.

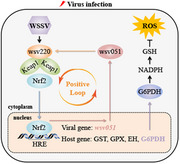

## 1 Introduction

Reactive oxygen species (ROS) refers to a set of reactive molecules originating from molecular oxygen and generated as products of host oxygen metabolism.^[^ ^1^, ^2^ ^]^ ROS is essential for the functioning of biological systems and serves as signaling molecules that regulate various essential cellular functions.^[^ ^4^ ^]^ Maintaining ROS homeostasis is a fundamental prerequisite for biological systems to carry out various essential cellular functions, such as cellular growth, differentiation, aging, and survival in hosts.^[^ ^3^, ^4^ ^]^ The generation of ROS can be induced by a multitude of stresses, such as pathogenic infections.^[^ ^5^, ^6^, ^7^ ^]^ As an instance, the Hepatitis C virus (HCV) core protein NS5A causes mitochondrial dysfunction due to Ca^2+^ redistribution, resulting in increased ROS levels.^[^ ^8^ ^]^ Likewise, the Hepatitis B virus X protein promotes ROS generation by binding to voltage‐dependent anion channel 3, a component of the mitochondrial permeability transition pore.^[^ ^9^ ^]^ Infectious salmon anemia virus induces ROS production via nicotinamide adenine dinucleotide phosphate (NADPH) oxidase complex activation in a p38 MAPK‐dependent manner in *Salmo salar*, while Spring viraemia of carp virus disrupts mitochondrial complex III, leading to ROS accumulation.^[^ ^10^ ^]^ However, ROS assumes a dual role in host defense against pathogens. On one hand, ROS functions as an integral component of the innate immune response.^[^ ^11^, ^12^ ^]^ In the context of viral infection, the production of ROS helps to create an oxidative environment that is detrimental to the virus, inhibiting viral replication and preventing the spread of the infection.^[^ ^11^, ^13^, ^14^, ^15^ ^]^ ROS can also activate immune cells such as macrophages and neutrophils, or trigger immune signaling pathways via various mechanisms, which can further contribute to the elimination of the virus.^[^ ^2^ ^]^ On the other hand, excessive or uncontrolled ROS production causes oxidative damage to the host cellular components including proteins, lipids, and DNA, triggers severe inflammatory responses, and even leads to cell death.^[^ ^3^ ^]^ This oxidative stress impairs cellular function and contributes to the host's susceptibility to infection.^[^ ^16^ ^]^ Hence, maintaining a dynamic balance of ROS production and its scavenging is key to both host and virus.

In response, some viruses have evolved mechanisms to modulate host ROS dynamics ensuring their survival and propagation.^[^ ^17^, ^18^, ^19^, ^20^, ^21^, ^22^ ^]^ For example, the Kaposi's sarcoma‐associated herpesvirus activates the nuclear factor erythroid 2‐related factor 2 (Nrf2) pathway to enhance antioxidant responses and create a microenvironment conducive to infection.^[^ ^16^ ^]^ The human cytomegalovirus upregulates antioxidant enzyme expression to counteract ROS‐induced damage during infection.^[^ ^23^ ^]^ This modulation of ROS by viruses is not merely a byproduct of infection but a critical component of viral pathogenesis, influencing disease outcomes. It is of crucial importance to characterize ROS dynamics and reveal ROS homeostatic mechanisms during viral infection. Nrf2 has been recognized as the core regulator of ROS homeostasis.^[^ ^24^, ^25^ ^]^ The Nrf2 pathway regulates antioxidant genes and phase II detoxification enzymes to maintain cellular redox balance.^[^ ^24^, ^26^ ^]^ Under normal physiological conditions, Nrf2 interacts with its repressor, Kelch‐like ECH‐associated protein 1 (Keap1), via its two highly conserved motifs, “ETGE” and “DLG”, leading to the rapid degradation of Nrf2 by proteasomes.^[^ ^26^ ^]^ However, under oxidative stress, the inhibition of Keap1 on Nrf2 is useless since their binding is destroyed. The subsequent translocation of Nrf2 into the nucleus facilitates its binding to the antioxidant response element (ARE), promoting the transcription of antioxidant and detoxifying genes.^[^ ^24^ ^]^ Importantly, many viruses have evolved mechanisms to target the Nrf2 pathway, regulating redox homeostasis and contributing to viral pathogenesis and progression.^[^ ^27^, ^28^, ^29^ ^]^ Despite significant progress in understanding viral mechanisms that stimulate ROS production, the exact strategies by which viruses control ROS or mitigate their detrimental effects remain an area of active investigation.^[^ ^17^ ^]^

White spot syndrome virus (WSSV), the sole member of the genus Whispovirus in the family *Nimaviridae*, is an enveloped dsDNA virus that extensively infects crustaceans.^[^ ^30^, ^31^ ^]^ WSSV is prevalent in farmed shrimp globally and causes significant economic losses in prawn aquaculture annually.^[^ ^32^, ^33^ ^]^ Previous studies have shown that WSSV infection induces excessive ROS and oxidative stress, benefiting the host by limiting viral replication.^[^ ^34^, ^35^, ^36^ ^]^ Notably, ROS levels increase sharply from 0.5 to 2 hours post‐infection (hpi) in shrimp *Litopenaeus vannamei* (*L. vannamei*), but return to basal levels within 6 h.^[^ ^35^ ^]^ This rapid decline suggests that WSSV has mechanisms to counteract the host's ROS defense. Furthermore, WSSV has been shown to restore cellular redox balance by enhancing the activity of SOD and GPx,^[^ ^37^ ^]^ which are downstream Nrf2 target genes.^[^ ^38^, ^39^ ^]^ These observations imply a potential role for the Nrf2 pathway in mitigating WSSV‐induced oxidative stress and supporting viral replication, though the precise mechanisms remain unclear.

Here, we discovered that the WSSV protein wsv220 directly binds to the host *L. vannamei* Nrf2 (LvNrf2) repressor LvKeap1, disrupting the LvNrf2/LvKeap1 complex and promoting Nrf2 nuclear translocation. LvNrf2 then induces the expression of downstream glucose‐6‐phosphate dehydrogenase (LvG6PDH), counteracting excessive ROS by increasing NADPH and glutathione (GSH) production, which inhibit ROS. Interestingly, LvNrf2 also triggers the expression of the WSSV gene wsv051, elevating wsv220 expression and creating a positive feedback loop that continuously facilitates WSSV replication. Our results reveal a novel molecular mechanism by which WSSV hijacks the Nrf2/Keap1 antioxidant system to eliminate cellular ROS and promote virus proliferation.

## 2 Results

### 2.1 WSSV Infection Regulated ROS and Activated the LvNrf2 Pathway

**Figure 1:**
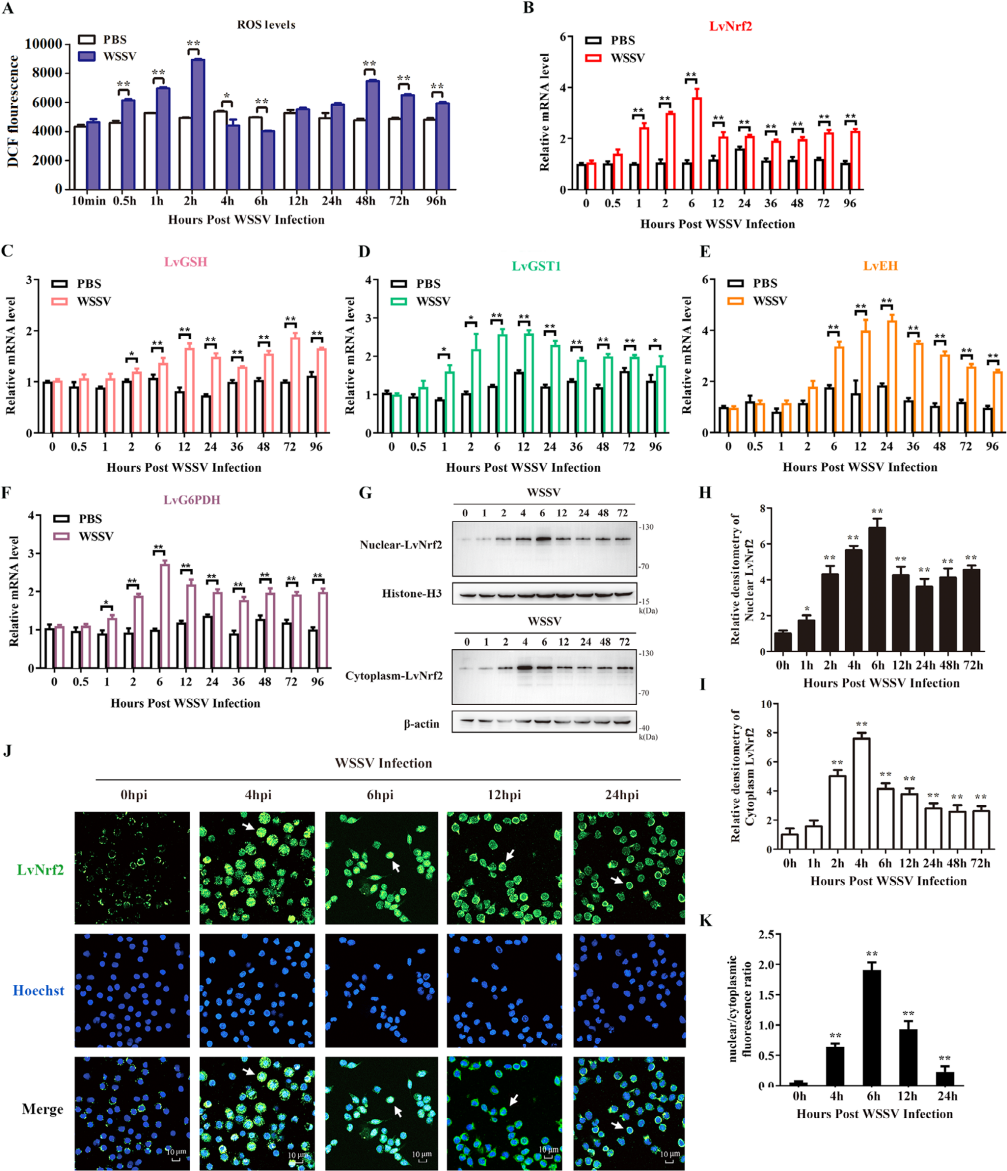
WSSV infection reduces ROS levels and activates the LvNrf2 pathway. A) Dynamic changes in ROS levels in hemocytes following WSSV infection. B–F) Expression profiles of LvNrf2 (B) and its target genes LvGSH (C), LvGST1 (D), LvEH (E), and LvG6PDH (F) in hemocytes after WSSV or PBS (control) injection, assessed by qPCR. G) LvNrf2 expression in the nucleus and cytoplasm following WSSV infection, detected by western blotting. H,I) Statistical analysis of LvNrf2 levels in the nucleus (H) and cytoplasm (I) using WCIF ImageJ software, corresponding to (G). J) LvNrf2 nuclear translocation in response to WSSV infection. Hemocytes were collected at 0, 4, 6, 12, and 24 hpi and subjected to immunofluorescence staining, visualized by confocal laser scanning microscopy. K) The ratio of cytoplasm/nuclear LvNrf2 shown in Figure 1J, calculated using WCIF ImageJ software and statistically analyzed by Student's *t‐*test (^**^ *p* < 0.01, ^*^ *p* < 0.05).

To investigate ROS response to WSSV infection, we monitored the dynamic changes of ROS after infection. ROS levels were higher than the control from 0.5 to 2 hpi and 48 to 96 hpi but lower than the control from 4 to 6 hpi (**Figure** **1**A). The ratio of NADPH/NADP^+^ was lower than the control at 2 hpi, and the ratios of NADPH/NADP^+^ and GSH/GSSH were higher than the control at 6 to 12 hpi (Figure S1A,B, Tables S2 and S3, Supporting Information). These results indicate that WSSV modulates host ROS levels.

To assess the impact of WSSV infection on the Nrf2 pathway, we cloned and identified LvNrf2 (XM_07 014 0254.1), which contains 788 amino acids and is translated from a 2364 nucleotide (nt) mRNA species with a 289 nt 5′‐UTR and a 558 nt 3′‐UTR (Figure S2, Supporting Information). Phylogenetic analysis showed that Nrf2 from invertebrates, including *L. vannamei* and insects, clustered together (Figure S3, Supporting Information). The transcription levels of LvNrf2 increased from 1 hpi to 96 hpi in hemocytes after WSSV challenge (Figure 1B). Correspondingly, the transcription levels of LvNrf2 target genes increased during WSSV infection (Figure 1C–F). Specifically, the expression levels of glutathione S‐transferase (LvGST1, XM_02 738 2382.2) and LvG6PDH (MK814533.1) were elevated from 1 to 96 hpi. And, glutathione peroxidase (LvGSH, XM_02 736 8747.2) showed increased expression from 2 to 96 hpi, epoxide hydrolase (LvEH, XM_02 736 9996.2) showed increased expression from 6 to 96 hpi. The expression of LvNrf2 in both the nucleus and cytoplasm increased from 2 hpi (Figure 1G–I). Immunofluorescence assays showed enhanced translocation of LvNrf2 to the nucleus at 4–24 hpi (Figure 1J,K), while its cytoplasmic distribution also increased from 4 hpi (Figure 1J). Thus, WSSV increases the expression of LvNrf2 in the cytoplasm and promotes its nuclear translocation, demonstrating that WSSV activates the LvNrf2 pathway.

### 2.2 LvNrf2 Facilitated WSSV Replication and Reduced ROS

**Figure 2:**
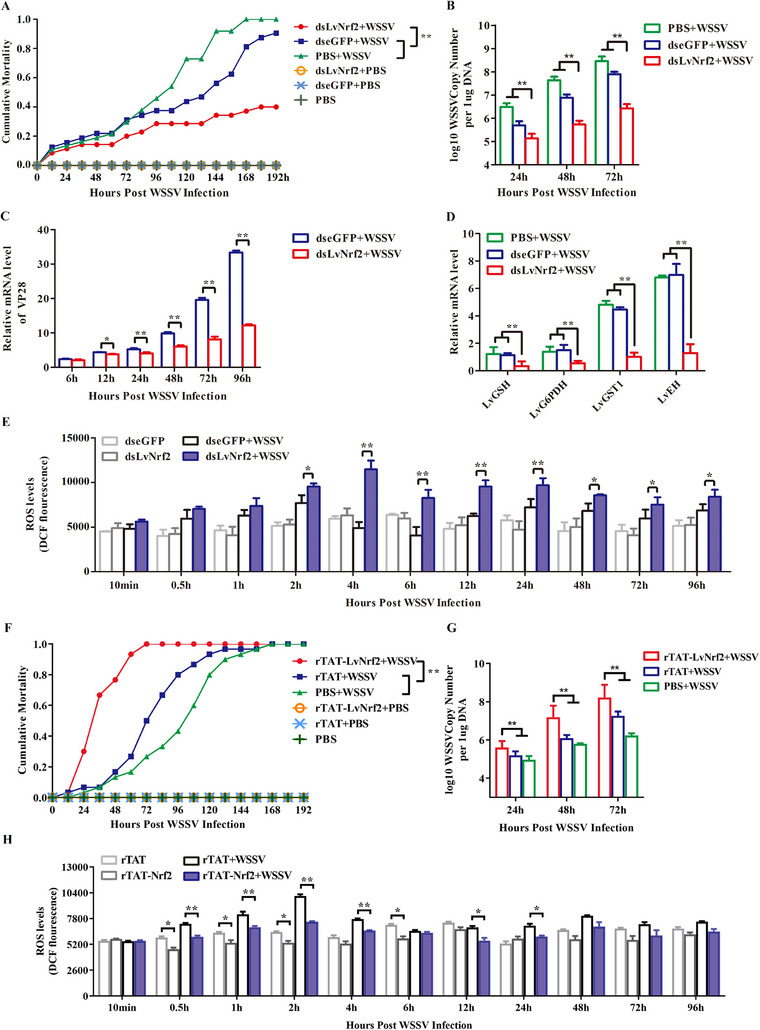
LvNrf2 facilitates WSSV replication and reduces ROS. A) LvNrf2 knockdown enhances resistance to WSSV infection. WSSV was inoculated 48 h post‐dsLvNrf2 injection, and shrimp mortality was recorded every 8 h to analyze cumulative mortality. B) LvNrf2 knockdown results in reduced WSSV genome replication levels in muscle tissue. Viral load was assessed at 24 h, 48 h, and 72 h post‐dsLvNrf2 injection through absolute qPCR. C) The mRNA level of VP28 in hemocytes of dsRNA‐treated shrimp after WSSV injection was detected by qPCR. D) mRNA levels of LvGSH, LvG6PDH, LvGST1, and LvEH in RNAi‐treated shrimp after WSSV or PBS injection in hemocytes, assessed by qPCR. E) ROS levels in hemocytes of dsLvNrf2‐injected shrimp after WSSV or PBS injection. F) rTAT‐LvNrf2‐injection decreases the resistance of shrimp to WSSV infection. WSSV was inoculated 48 h post‐rTAT‐LvNrf2 injection, and shrimp mortality was recorded as above. G) rTAT‐LvNrf2‐injection results in increased WSSV replication levels in muscle tissue. Viral load was assessed as above. H) ROS levels in hemocytes of rTAT‐LvNrf2‐ injected shrimp after WSSV or PBS injection. Data were statistically analyzed by Student's *t‐*test (^**^ *p* < 0.01, ^*^ *p* < 0.05).

An RNAi experiment was performed to knock down LvNrf2 expression in vivo. RNAi efficiency was assessed by qPCR and western blotting at 48 h post‐dsRNA injection, showing a significant reduction in LvNrf2 gene transcription and protein levels (Figure S4A, Supporting Information). RNAi‐treated shrimp were challenged with WSSV, and WSSV copies were analyzed by absolute quantitative PCR. The mortality of dsLvNrf2‐treated shrimp post‐WSSV infection (40%) was lower than that of dseGFP‐treated shrimp (90.6%) at 192 hpi (**Figure** **2**A). LvNrf2 knockdown decreased WSSV genome replication (Figure 2B), the expression of VP28 (Figure 2C), and the expression of LvNrf2 target genes (LvGSH, LvGST1, LvEH, and LvG6PDH) (Figure 2D). ROS levels increased and were maintained at higher levels from 2 to 96 hpi in dsLvNrf2‐treated shrimp (Figure 2E). Correspondingly, the ratios of NADPH/NADP^+^ and GSH/GSSH decreased due to LvNrf2 knock down (Figure S4E,F, Supporting Information), leading to a loss of suppression of ROS production. Recombinant TAT‐connected LvNrf2 protein (rTAT‐LvNrf2) was injected into shrimp to overexpress LvNrf2 in vivo (Figure S6A, Supporting Information), and rTAT protein was injected as a control (Figure S6B, Supporting Information). Injection of LvNrf2 increased the expression of LvNrf2 in hemocytes but did not affect the expression of LvKeap1 (Figure S4B–D, Supporting Information). LvNrf2 overexpression led to 100% mortality within 72 hpi (Figure 2F) and increased viral loads during WSSV infection (Figure 2G). The ROS levels in rTAT‐LvNrf2‐treated WSSV‐challenged shrimp decreased at 0.5–24 hpi (Figure 2H), and the ratios of NADPH/NADP^+^ and GSH/GSSH increased from 2 to 6 hpi (Figure S4G,H, Supporting Information).

Another RNAi experiment was conducted to knock down LvKeap1 (XM_07 014 1304.1) expression to activate LvNrf2 in vivo. DsKeap1 injection significantly decreased LvKeap1 expression in both mRNA and protein levels, leading to an increased LvNrf2 protein expression without significantly altering LvNrf2 mRNA levels (Figure S5A,B, Supporting Information). After WSSV challenge, LvNrf2 expression increased in both nucleus and cytoplasm of dsLvKeap1‐treated shrimp (Figure S5C–F, Supporting Information). Higher mortality was observed in dsLvKeap1‐treated shrimp post‐WSSV infection (100%) compared to the dseGFP‐treated control group (75%) at 108 hpi, as well as viral loads increased after dsLvKeap1 treated (Figure S5G,H, Supporting Information). The ROS levels in dsLvKeap1‐treated WSSV‐challenged shrimp significantly decreased at 0.5–2 hpi and 24–96 hpi (Figure 5I), while the ratios of NADPH/NADP^+^ and GSH/GSSH increased from 1 to 4 hpi (Figure S5J,K, Supporting Information). These data strongly demonstrate that the Nrf2 pathway contributes to WSSV infection and reduces WSSV‐induced ROS.

### 2.3 LvNrf2 Inhibited ROS by Inducing LvGSH, LvG6PDH, LvEH, and LvGST1

**Figure 3:**
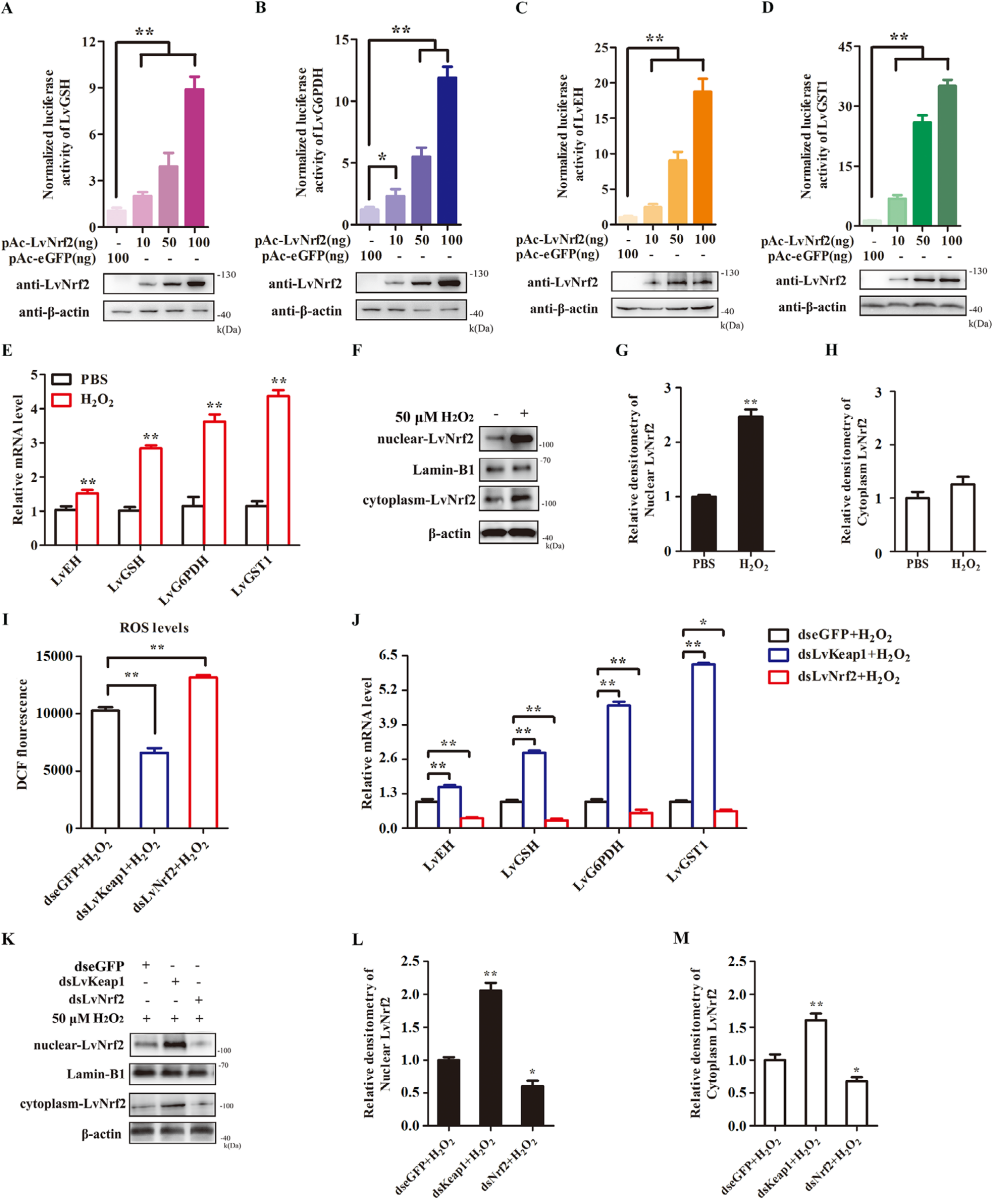
LvNrf2 inhibits ROS by inducing LvGSH, LvG6PDH, LvEH, and LvGST1. A–D) Effects of LvNrf2 on the promoter activities of LvGSH (A), LvG6PDH (B), LvGST1 (C), and LvEH (D) in *Drosophila* S2 cells. E) Transcription levels of LvEH, LvGSH, LvG6PDH, and LvGST1 were detected by qPCR at 48 h after H_2_O_2_ injection. F–H) Expression levels of LvNrf2 in the nucleus (G) and cytoplasm (H) were detected at 48 h after H_2_O_2_ injection by western blotting. I) ROS levels in hemocytes after LvNrf2 or LvKeap1 knockdown. J) Expression levels of LvNrf2 target genes (LvEH, LvGSH, LvG6PDH, and LvGST1) in hemocytes from dsLvNrf2, dsLvKeap1, or dseGFP‐treated. K–M) Expression levels of LvNrf2 in the nucleus (L) and cytoplasm (M), along with statistical analysis by WCIF ImageJ software, corresponding to (K) in dsRNA‐injected shrimp followed by H_2_O_2_ treatment at 48 h. Data were statistically analyzed by Student's *t‐*test (^**^ *p* < 0.01, ^*^ *p* < 0.05).

To verify the function of Nrf2‐dependent cytoprotective genes in mitigating oxidative stress, expression plasmids of LvNrf2 were co‐transfected with luciferase reporter plasmids of cytoprotective genes into *Drosophila* S2 cells, including LvGSH, LvG6PDH, LvEH, and LvGST1. The results showed that LvNrf2 upregulated the promoter activity of LvGSH, LvG6PDH, LvEH, and LvGST1 in a concentration‐dependent manner (**Figure** **3**A–D). To further verify the ability of the LvNrf2 pathway to eliminate ROS, H_2_O_2_ was used to induce oxidative stress in hemocytes. ROS levels increased after 50 µm H_2_O_2_ injection (Figure S7A, Supporting Information). H_2_O_2_ stimulation elevated the transcription levels of LvNrf2 target genes LvEH, LvGSH, LvG6PDH, and LvGST1 (Figure 2E), and increased nuclear accumulation of LvNrf2 (Figure 2F–H). These results indicate that LvNrf2 responds to H_2_O_2_‐induced oxidative stress. RNAi experiments were performed to knockdown LvNrf2 or LvKeap1 followed by H_2_O_2_ treatment. The gene‐specific dsRNAs efficiently inhibited the expression of LvNrf2 or LvKeap1 (Figure S7B,C, Supporting Information). After H_2_O_2_ treatment, ROS levels decreased in dsLvKeap1‐treated shrimp at 48 h (Figure 3I). The transcriptional levels of LvEH, LvGSH, LvG6PDH, and LvGST1 increased in dsLvKeap1‐treated shrimp (Figure 3J), as did the expression of LvNrf2 in both nucleus and cytoplasm (Figure 3K–M). These data indicate that LvNrf2 regulates ROS by promoting the expression of antioxidant genes, consistent with observations in dsLvNrf2‐treated H_2_O_2_‐injected shrimp (Figure 3I–M).

### 2.4 Wsv220 Activated the LvNrf2 Pathway

**Figure 4:**
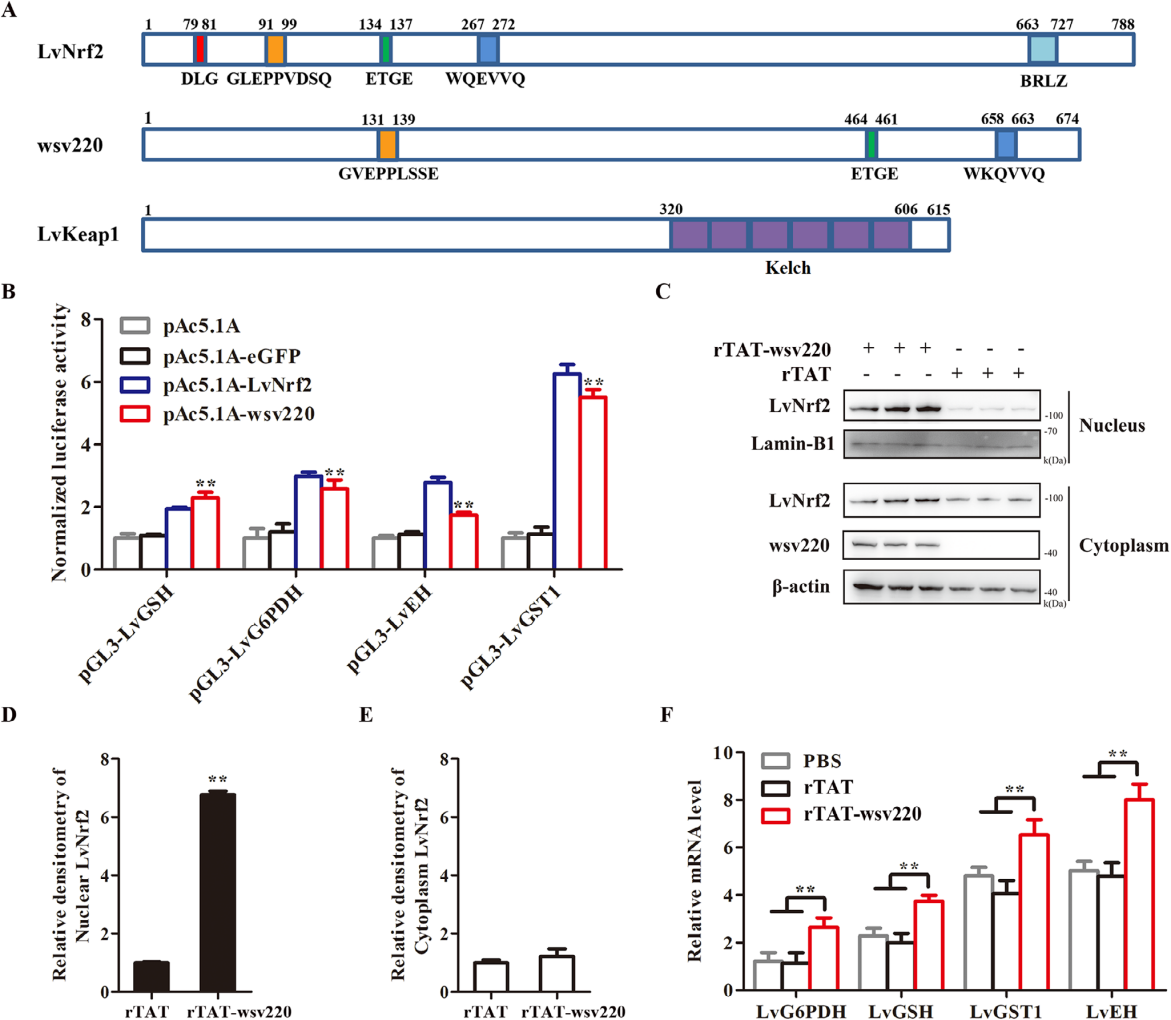
wsv220 activates the LvNrf2 pathway. A) Schematic diagram of the domain structures of LvNrf2, wsv220, and LvKeap1. B) Effects of wsv220 overexpression on the promoter activities of LvGSH, LvG6PDH, LvEH, and LvGST1 in *Drosophila* S2 cells. C–F) Protein levels of LvNrf2 (D), and (F) transcription levels of LvG6PDH, LvGSH, LvGST1, and LvEH after injecting purified rTAT‐wsv220‐GST protein in vivo. Statistical analysis of LvNrf2 levels in the nucleus (D) and cytoplasm (E) using WCIF ImageJ software corresponding to (C). Data were statistically analyzed by Student's *t‐*test (^**^ *p* < 0.01, ^*^ *p* < 0.05).

In order to explore the molecular mechanism of WSSV activating Nrf2 pathway, we found an “ETGE” motif in wsv220 (NP_477 742.1) by biogenic analysis of WSSV sequences. Wsv220 is a viral nucleocapsid protein with a predicted molecular mass of 76 kDa.^[^ ^40^ ^]^ The “ETGE” motifs in the Neh2 region of Nrf2 are necessary for the binding of Nrf2 and Keap1.^[^ ^24^ ^]^ We speculated that wsv220 might activate Nrf2 by binding to Keap1. Additionally, wsv220 has two Nrf2 homologous sites, “^131^GVEPPLSSE^139^” and “^658^WKQVVQ^663^” (**Figure** **4**A). We speculated that wsv220 might activate Nrf2 by binding to Keap1. Owing to the unavailability of culturable shrimp cell lines, we utilized 293T cells as a surrogate model to investigate the impact of wsv220 on endogenous Nrf2 expression. The interaction between human Nrf2 (hNrf2) and human Keap1 (hKeap1) has been confirmed through Co‐immunoprecipitation (Co‐IP) assays in 293T cells (Figure S8A, Supporting Information). Overexpression of wsv220 in 293T cells increased the accumulation of hNrf2 in the nucleus and did not affect the expression of hKeap1 (Figure S8B, Supporting Information). A luciferase reporter gene assay further showed that wsv220 upregulates the promoter activities of LvGSH, LvG6PDH, LvEH, and LvGST1 in *Drosophila* S2 cells (Figure 4B). Recombinant GST‐tagged TAT‐connected wsv220 protein (rTAT‐wsv220‐GST) or rTAT‐GST (control) was injected into shrimp to overexpress wsv220 or GST in vivo (Figure S6C,D, Supporting Information). Injection of wsv220 protein increased the accumulation of LvNrf2 in both cytoplasm and nucleus (Figure 4C–E) but did not affect the expression of LvKeap1 (Figure S8C, Supporting Information), and increased the transcription levels of LvG6PDH, LvGSH, LvGST1, and LvEH (Figure 4F). These results indicate that wsv220 activates the Nrf2 pathway.

### 2.5 Competitive Binding of Wsv220 or LvNrf2 to LvKeap1

**Figure 5:**
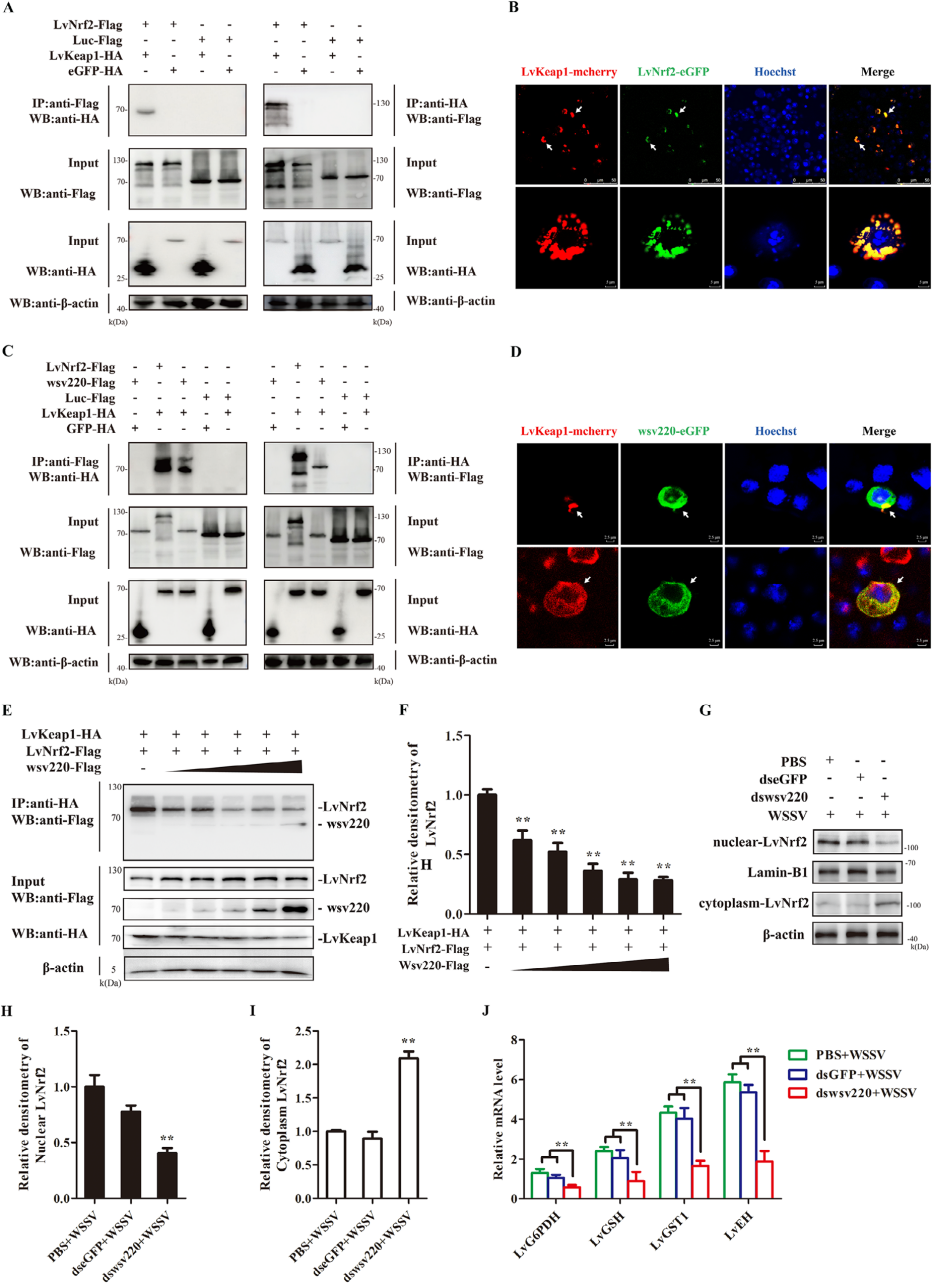
wsv220 competes with LvNrf2 for binding to LvKeap1. A) Reciprocal immunoprecipitations of Nrf2 and Keap1 were performed on *Drosophila* S2 cells co‐transfected with plasmids pAc5.1A‐LvNrf2‐Flag and pAc5.1A‐LvKeap1‐HA. 24 h post‐transfection, cells were collected and subjected to Co‐IP with Flag or HA beads. pAc5.1A‐Luc‐Flag and pAc5.1A‐eGFP‐HA were used as negative controls. B) Immunofluorescence assay to analyze the interactions between Nrf2 and Keap1 in *Drosophila* S2 cells, observed with laser confocal microscopy at 24 h post‐co‐transfection with pAc5.1A‐LvNrf2‐eGFP and pAc5.1A‐LvKeap1‐mCherry. Red fluorescence represents LvKeap1, green fluorescence represents LvNrf2, and nuclei were stained with DAPI (blue). C,D) Interaction between wsv220 and LvKeap1 identified by Co‐IP (C) and immunofluorescence assays (D). E,F) Co‐IP analysis showed that wsv220 competes with LvNrf2 for binding LvKeap1 in *Drosophila* S2 cells (E). The interaction between LvNrf2 and LvKeap1 is gradually inhibited in a dose‐dependent manner by wsv220 (F). G) Expression of LvNrf2 in the nucleus and cytoplasm of dsRNA‐pretreated shrimp at 48 h after WSSV infection, detected by western blotting. H,I) Statistical analysis of LvNrf2 in the nucleus (H) and cytoplasm (I) by WCIF ImageJ software, corresponding to (G). J) Transcription levels of LvG6PDH, LvGSH, LvGST1, and LvEH were detected by qPCR in hemocytes of dsRNA‐pretreated shrimp at 48 h after WSSV injection. Data was statistically analyzed by Student's *t‐*test (^**^ *p* < 0.01, ^*^ *p* < 0.05).

To investigate the mechanism by which wsv220 activates the Nrf2 pathway, we verified the interactions between Keap1, Nrf2, and wsv220 by Co‐IP assays in *Drosophila* S2 cells. LvNrf2 (**Figure** **5**A) and wsv220 (Figure 5C) were able to interact with LvKeap1. When LvNrf2 or wsv220 co‐expressed with LvKeap1, LvNrf2 and LvKeap1, or wsv220 and LvKeap1 co‐localized in the cytoplasm of *Drosophila* S2 cells via immunofluorescence assays (Figure 5B–D). The co‐expression of LvNrf2 and wsv220 in *Drosophila* S2 cells served as a negative control (Figure S9, Supporting Information), demonstrating no detectable interaction. We then investigated if wsv220 disrupts the interaction between LvKeap1 and LvNrf2 by co‐expressing LvKeap1 and LvNrf2 with wsv220 in *Drosophila* S2 cells. The results showed that wsv220 inhibited LvNrf2 binding to LvKeap1 in a concentration‐dependent manner (Figure 5E,F). To provide further evidence of wsv220's ability to release LvNrf2 from Keap1, we investigated the impact of wsv220 on the activation of the LvNrf2 pathway in vivo. The RNAi experiments effectively reduced the expression of wsv220 (Figure S10A, Supporting Information). Suppression of wsv220 expression resulted in decreased expression of Nrf2 and impaired its nuclear migration post‐WSSV infection (Figure 5G–I), leading to reduced transcriptional levels of LvG6PDH, LvGSH, LvGST1, and LvEH (Figure 5J). Knockdown of wsv220 decreased viral loads and lowered mortality post‐WSSV infection (Figure S10B,C, Supporting Information). These results indicate that wsv220 activates LvNrf2 by competitively binding to LvKeap1.

### 2.6 Interaction Between Wsv220 and LvKeap1 via **“^464^ETGE^467^”, “^91^GVEPPLSSE^99^”, and “^658^WKQVVQ^663^” Regions**

**Figure 6:**
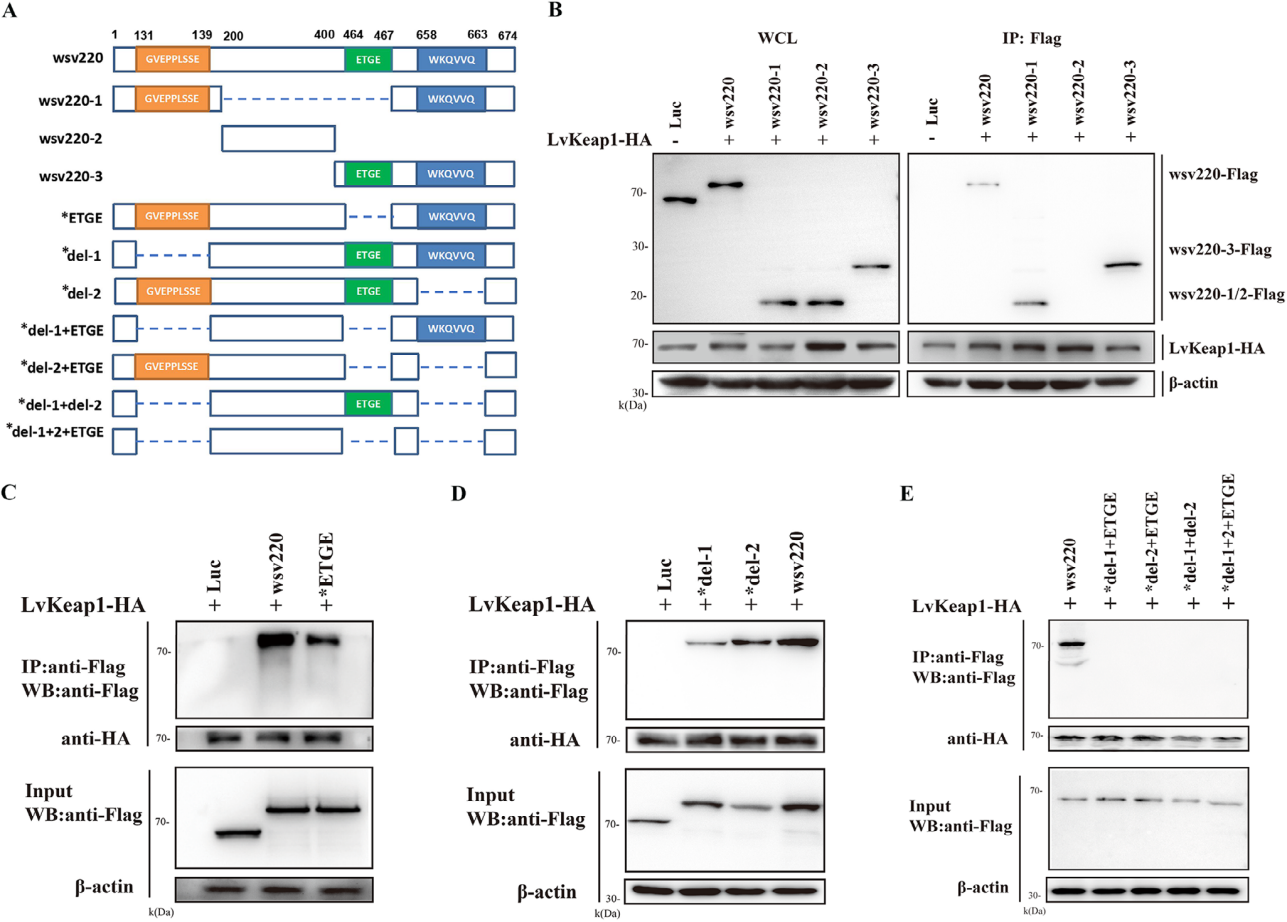
wsv220 interacts with LvKeap1 through its three LvNrf2 homologous sites. A) Schematic diagram of the wsv220 domain structure and deletion mutants. All wsv220 deletion mutants were Flag‐tagged at the N‐terminus. Luciferin (Luc) was used as a control. B) Co‐IP analysis of the interaction between wsv220‐1, wsv220‐3, wsv220‐2, and LvKeap1. C) Co‐IP analysis of the interaction between the ETGE motif deletion mutant of wsv220 and LvKeap1. D) Co‐IP analysis of the interaction between deletion mutants (*del‐1, *del‐2) and LvKeap1. E) Co‐IP analysis of the interaction between multiple deletion mutants (*del‐1+ETGE, *del‐1+del‐2, and *del‐1+del‐2+ETGE) and LvKeap1.

To identify the binding site between wsv220 and LvKeap1, wsv220 mutants were generated with specific alterations in the “^464^ETGE^467^”, “^91^GVEPPLSSE^99^”, and “^658^WKQVVQ^663^” regions (**Figure** **6**A). Three truncated segments of wsv220 (wsv220‐1, wsv220‐2, and wsv220‐3) were co‐expressed with LvKeap1 or Luc (control). Co‐IP analyses revealed interactions between wsv220‐1 or wsv220‐3 with LvKeap1 (Figure 6B). The absence of the “ETGE” motif impaired the interaction between wsv220 and LvKeap1 (Figure 6C). Further, we generated wsv220 mutants by deleting two homologous regions with LvNrf2 located at 131–139 aa and 658–663 aa, referred to as *del‐1 and *del‐2 (Figure 6A). The interaction between wsv220 and LvKeap1 was attenuated upon deletion of these two regions (Figure 6D). Co‐IP assays with four types of multi‐region deletion mutants of wsv220 revealed that the removal of any two or three regions (“^464^ETGE^467^”, “^91^GVEPPLSSE^99^”, and “^658^WKQVVQ^663^”) disrupted its interaction with Keap1 (Figure 6E). These data demonstrate the essential role of these regions within wsv220 in mediating Keap1 binding.

### 2.7 LvG6PDH Was Required for ROS Inhibition and WSSV Infection

**Figure 7:**
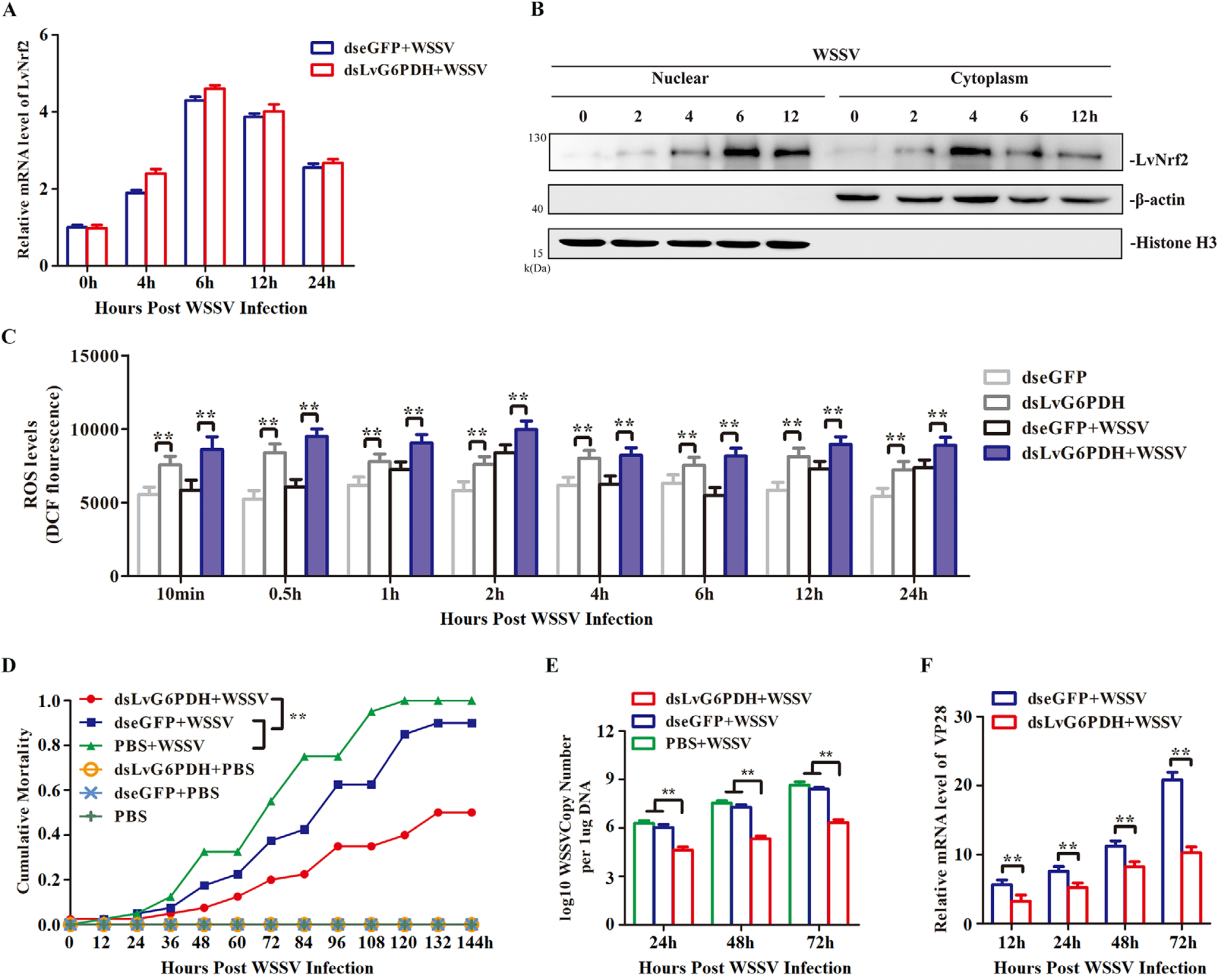
LvG6PDH is required for ROS inhibition and WSSV infection. A) Transcriptional levels of LvNrf2 in the gill of dsLvG6PDH‐treated, WSSV‐injected shrimp were detected by qPCR at 0, 4, 6, 12, and 24 hpi. B) Protein expression of LvNrf2 in hemocytes was detected at 0, 2, 4, 6, and 12 hpi by western blotting. C) Intracellular ROS levels in hemocytes of dseGFP‐ or dsLvG6PDH‐injected shrimp followed by WSSV challenge. D) Knockdown of LvG6PDH enhances resistance to WSSV infection. WSSV was inoculated 48 h post‐LvG6PDH knockdown, and shrimp mortality was recorded every 8 h to analyze cumulative mortality rates. E) Knockdown of LvG6PDH results in reduced WSSV replication levels in muscles. WSSV was inoculated 48 h post‐dsRNA injection, and viral load was assessed at 48 hpi by absolute qPCR. F) Expression level of VP28 detected in hemocytes of dsLvG6PDH‐treated shrimp after WSSV injection by qPCR. Data was statistically analyzed by Student's *t‐*test (^**^ *p* < 0.01, ^*^ *p* < 0.05).

G6PDH is the pivotal rate‐limiting enzyme in the pentose phosphate pathway (PPP), facilitating the conversion of glucose‐6‐phosphate from glycolysis into the PPP and catalyzing the transformation of NADP^+^ to NADPH.^[^ ^42^, ^43^ ^]^ NADPH donates electrons to reduce GSSG to GSH by glutathione reductase.^[^ ^42^ ^]^ We hypothesized that LvNrf2‐induced LvG6PDH is involved in WSSV infection by regulating NADPH and GSH generation. DsLvG6PDH was injected to suppress LvG6PDH expression followed by WSSV infection (Figure S11A, Supporting Information). The transcriptional level of LvNrf2 was not affected (**Figure** **7**A). The distribution of LvNrf2 in both cytoplasm and nucleus increased at 4 to 12 hpi (Figure 7B; Figure S11B,C, Supporting Information), indicating that WSSV activation of LvNrf2 is not affected by LvG6PDH. Knockdown of LvG6PDH suppressed the ratios of NADPH/NADP^+^ and GSH/GSSH post‐WSSV injection (Figure S11D,E, Supporting Information), leading to increased ROS levels (Figure 7C). The mortality of dsLvG6PDH‐treated post‐WSSV infection (50%) was lower than dseGFP‐treated shrimp (90%) (Figure 7D). Suppression of LvG6PDH decreased WSSV genome replication   and VP28 expression (Figure 7E,F). These results suggest that LvG6PDH is essential for ROS elimination during WSSV infection.

### 2.8 LvNrf2 Activates Wsv051 to Generate a Positive Feedback Loop that Facilitates WSSV Replication

**Figure 8:**
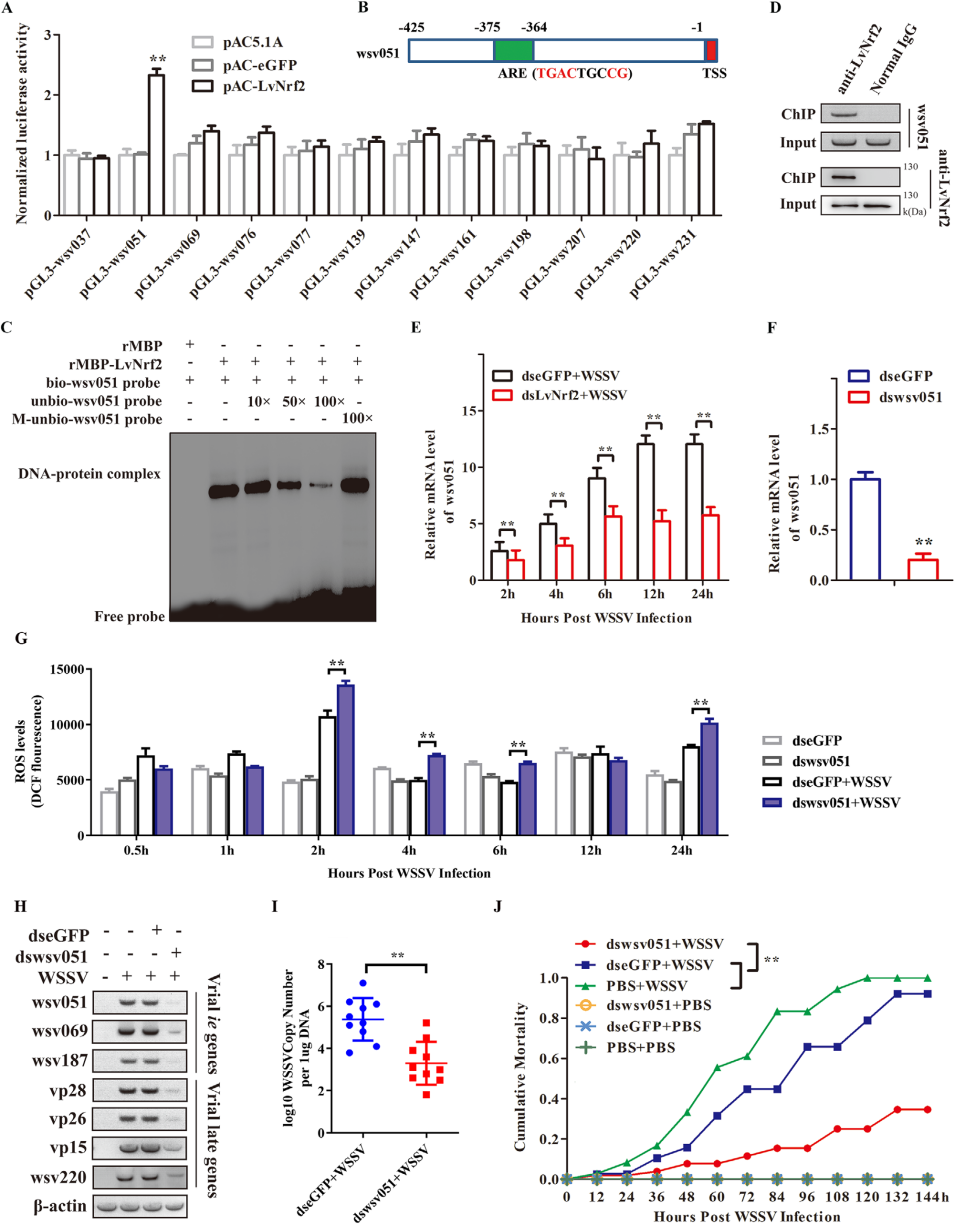
LvNrf2 activates wsv051 to create a positive feedback loop. A) Dual reporter assay screened the effects of LvNrf2 expression on the promoter activities of ARE motif‐containing WSSV genes in *Drosophila* S2 cells. B) Potential ARE motif in the promoter regions of wsv051. Putative ARE motifs are represented as blue rectangles, and TSS as red rectangles. C) EMSA assay verified the interaction between purified rMBP‐LvNrf2 and putative ARE motif of wsv051. D) ChIP assays analyzed the interaction between LvNrf2 and wsv051 in hemocytes of WSSV‐infected shrimp in vivo. E) Transcriptional levels of wsv051 in gills of dsNrf2‐ or dseGFP‐treated shrimp detected by qPCR during WSSV infection. F) Knockdown efficiencies of wsv051 in hemocytes assessed by qPCR at 48 h post‐dsRNA injection. G) Intracellular ROS levels in hemocytes of dseGFP‐ or dswsv051‐injected shrimp followed by WSSV challenge. H) Semi‐quantitative PCR quantified the expression of wsv051, wsv069, and wsv187, vp28, vp26, vp15, and wsv220 using β‐actin as a control. I) wsv051 knockdown lowered viral loads in muscle tissue. WSSV was inoculated 48 h post‐dsRNA injection, and viral load was assessed at 48 hpi by absolute qPCR. J) Suppression of wsv051 enhanced resistance to WSSV infection. Shrimp mortality was recorded every 8 h for cumulative mortality rate analysis. Data was statistically analyzed by Student's *t‐*test (^**^ *p* < 0.01, ^*^ *p* < 0.05).

By screening WSSV sequences, we identified 12 WSSV gene promoters harboring the putative ARE motif, which is the conserved transcription factor binding motif of Nrf2. The regulation of LvNrf2 on these viral genes was assessed using a dual‐luciferase assay. The promoter activity of wsv051 (NP_477 573.1) was significantly upregulated by LvNrf2 (**Figure** **8**A). The ARE motif (TGACTGCCG) in the promoter region of wsv051 was identified at −375 to −364 upstream of the transcription start site (TSS) (Figure 8B). An EMSA assay determined the potential interaction between the ARE motif of wsv051 and LvNrf2. The results showed that recombinant MBP‐tagged LvNrf2 protein (rMBP‐LvNrf2) could interact with the ARE motif of wsv051 (Figure 8C; Figure S6E,F, Supporting Information), while no binding band was observed in the case of rMBP (control). ChIP assays further demonstrated that LvNrf2 binds to wsv051 promoter in vivo (Figure 8D). The transcriptional level of wsv051 was extremely reduced in dsLvNrf2‐treated shrimp during WSSV infection (Figure 8E). These findings demonstrate the activation of wsv051 by Nrf2 both in vivo and in vitro. wsv051, one of the 21 identified immediate‐early genes (IE) genes, plays a critical role in promoting the expression of other WSSV genes and is advantageous to viral infection.^[^ ^41^ ^]^ To further investigate the impact of wsv051 on WSSV replication, semi‐quantitative PCR analysis was conducted to detect viral gene transcription treated with dswsv051 following WSSV infection. Wsv051 expression was efficiently reduced after dsRNA injection (Figure 8F). Knockdown of wsv051 suppressed the ratios of NADPH/NADP+ and GSH/GSSH post‐WSSV injection (Figure S12A,B, Supporting Information), leading to increased ROS levels (Figure 8G). Knockdown of wsv051 suppressed the transcription of WSSV genes, including wsv069, wsv187, vp28, vp26, and wsv220 (Figure 8H). Notably, the expression of wsv220 was also decreased in dswsv051‐injected shrimp. This indicates that wsv051 elevates wsv220 expression, which further activates LvNrf2. A positive feedback loop of wsv220‐Nrf2‐wsv051 is established to facilitate WSSV replication. Disruption of this loop by knockdown of wsv051 resulted in a reduction in viral loads and mortality compared to the control following WSSV infection (Figure 8I,J). In summary, we concluded that WSSV hijacks the LvNrf2 pathway via wsv220 to suppress excessive ROS production in the host, creating a positive feedback loop to facilitate virus replication.

### 2.9 LvKelch Injection Inhibits WSSV Infection via Inducing Higher ROS Levels

**Figure 9:**
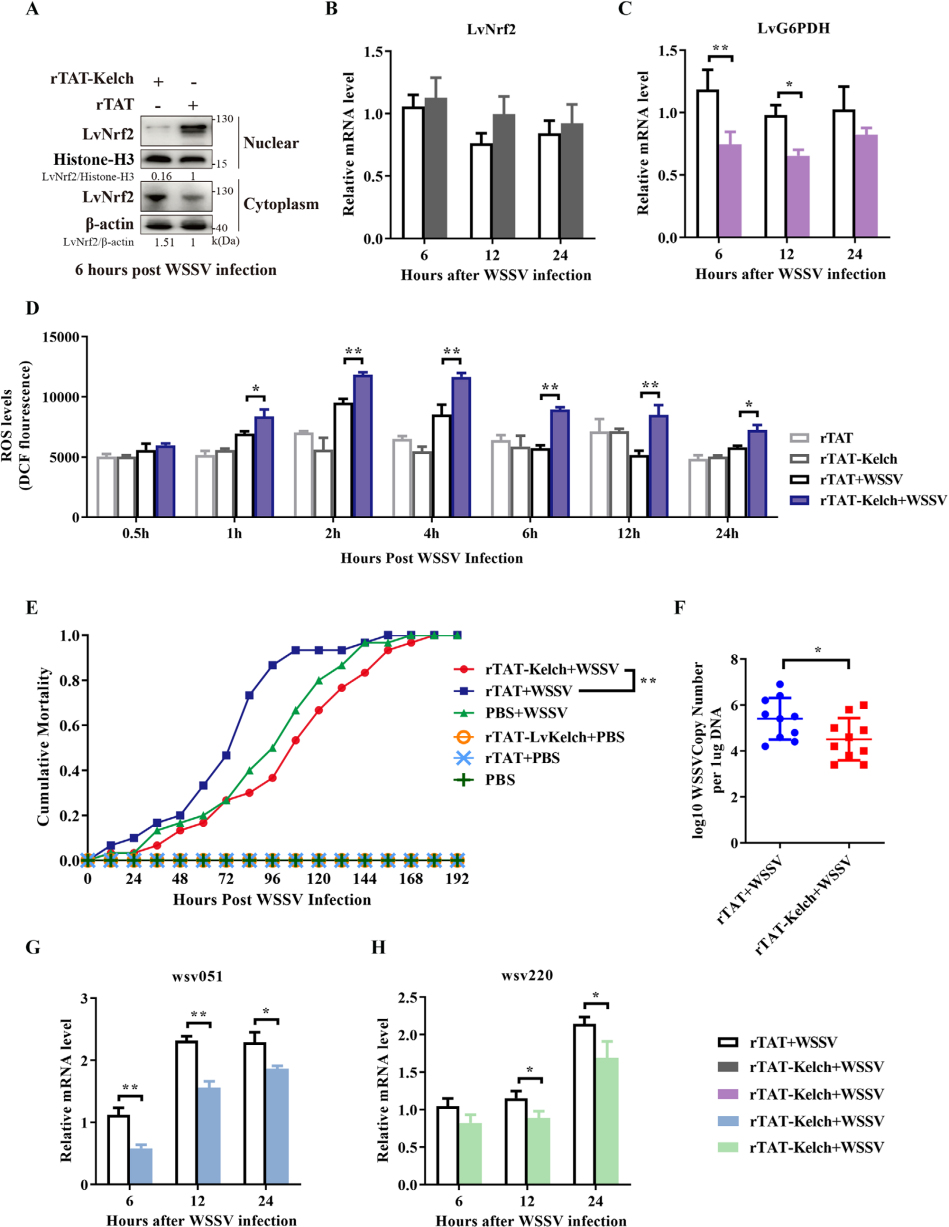
LvKelch injection inhibits WSSV infection via inducing higher ROS levels. A) Protein expression of LvNrf2 in nuclear and cytoplasm of hemocytes was detected at 6 hpi by western blotting. B,C) Transcription levels of LvNrf2 (B) and LvG6PDH (C) in hemocytes were detected at 6, 12 and 24 hpi by qPCR. D) Intracellular ROS levels in hemocytes of rTAT‐ or rTAT‐LvKelch‐ injected shrimp followed by WSSV infection. E) LvKelch injection enhances the resistance of shrimp to WSSV infection. F) LvKelch injection results in reduced WSSV replication levels in muscles. G,H) Transcription levels of wsv051 (G) and wsv220 (H) in hemocytes were detected at 6, 12, and 24 hpi by qPCR. Data were statistically analyzed by Student's *t‐*test (^**^ *p* < 0.01, ^*^ *p* < 0.05).

The Kelch domain of Keap1 is the conserved site for binding Nrf2. The interaction between Kelch domain of LvKeap1 (LvKelch) and LvNrf2 has been validated through Co‐IP (Figure S13A, Supporting Information). Is it feasible to inhibit the WSSV‐induced regulation of host ROS levels by sequestering Nrf2 in cytoplasm via the injection of LvKelch? Recombinant His‐tagged TAT‐connected LvKelch protein (rTAT‐LvKelch‐His) was injected into shrimp to overexpress LvKelch in vivo (Figure S6G, Supporting Information), rTAT‐His injection was as control (Figure S6B, Supporting Information). Injection of LvKelch protein decreased the accumulation of LvNrf2 in nucleus but increased its in cytoplasm (Figure S13B, Supporting Information), and did not affect the expression of LvKeap1 (Figure S13C,D, Supporting Information). During WSSV infection, LvKelch injection decreased the nuclear accumulation of LvNrf2 and the transcription of LvG6PDH, while did not affect the transcription of LvNrf2 (**Figure** **9**A‐C). The ROS levels in LvKelch‐treated WSSV‐challenged shrimp significantly decreased from 1 to 24 hpi (Figure 9D), and the ratios of NADPH/NADP^+^ and GSH/GSSH increased from 21 to 6 hpi (Figure S13E,F, Supporting Information). Notably, LvKelch injection increased the resistance of shrimp to WSSV (Figure 9E) and decreased viral loads (Figure 9F). And the transcription of wsv220 and wsv051 decreased in LvKelch‐treated WSSV‐challenge shrimp (Figure 9G,H). These results suggested that the injection of LvKelch suppresses WSSV replication by inhibiting the nuclear translocation of LvNrf2 and consequently preventing Nrf2 from modulating host ROS.

## 3 Discussion

Host produces ROS as a defense mechanism against viral invasion. Excessive ROS induce severe oxidative stress, resulting in detrimental effects on host cells while simultaneously eradicating the virus.^[^ ^17^ ^]^ Escape from ROS defense of host is crucial for viruses to achieve successful infection. Our findings demonstrate that WSSV exploits the host Nrf2 pathway to attenuate host ROS production and facilitate the successful completion of its replication cycle.

Given the detrimental consequences of excessive ROS production on viruses,^[^ ^11^ ^]^ viruses have naturally evolved mechanisms to control ROS or counteract ROS‐induced adverse effects to maintain a favorable cellular environment for viral replication.^[^ ^17^ ^]^ Increasing cellular antioxidant capacity is an effective strategy for viruses to directly eliminate excessive ROS. For instance, Cells exhibit elevated expression and activity of catalase upon transfection with human papillomavirus E7 protein, thereby conferring reduced susceptibility to H_2_O_2_.^[^ ^45^ ^]^ Similarly, the HCV protein NS5A activates Mn‐SOD through up‐regulating p38 MAPK, JNK, and AP‐1.^[^ ^46^ ^]^ HCV also induces the expression of GPx4 to inhibit the assistance of lipid peroxides in the formation of new virions.^[^ ^47^ ^]^ WSSV increases total antioxidant capacity and the activities of four antioxidant enzymes in different shrimp tissues, including catalase, peroxidase, superoxide dismutase, and thioredoxin peroxidase.^[^ ^48^ ^]^ Here, we first clone the full length of LvNrf2, containing conservative “DLG” motif, “ETGE” motif, and basic region leucin zipper (BRLZ) domain, which is more complete than previously reported Nrf2 in *L. vannamei* that only includes BRLZ domain.^[^ ^49^ ^]^ We demonstrate that WSSV activates Nrf2, thereby facilitating the restoration of redox homeostasis through modulation of the host antioxidant system. This well explains the previously observed enhancement in total antioxidant capacity and antioxidant enzyme activities after infection, exploited by WSSV to alleviate host ROS stress. This finding can be valuable for developing antiviral therapies that target the Nrf2 pathway or enhance its activation to boost the host's defense against WSSV.

Alternatively, viruses exert indirect control over the production of ROS by modulating metabolic pathways in host cells. Metabolic reprogramming is a pivotal strategy to ensure adequate supplies of molecules and energy required for viral replication and assembly. A common consequence of the virus infection is the induction of changes in glucose and lipid metabolism, leading to the occurrence of aerobic glycolysis known as the Warburg effect, and alters fatty acid utilization.^[^ ^50^, ^51^ ^]^ The metabolic shift to aerobic glycolysis prevents ROS overproduction by redirecting fuel away from oxidative phosphorylation.^[^ ^17^ ^]^ For example, the inhibition of lactate dehydrogenase A, a glycolytic enzyme, results in an elevated amount of pyruvate and heightened oxidative stress.^[^ ^52^ ^]^ Additionally, the key glycolytic enzyme phosphofructokinase 2 increases the glycolytic flux to counteract oxidative stress through an AMPK‐dependent manner in response to sublethal doses of H_2_O_2._^[^ ^53^ ^]^ A recent study reveals that WSSV enhances host glycolysis, and the accumulation of lactate promotes site‐specific histone lactylation to promote viral infection.^[^ ^54^ ^]^ WSSV also modulates the host lipid metabolism to damage host exoskeleton integrity.^[^ ^55^ ^]^ Suppression of the PI3K‐Akt‐mTOR pathway, which is crucial to the WSSV‐induced Warburg effect, leads to higher ROS production and lower WSSV genome replication.^[^ ^35^ ^]^ Here, we demonstrate that wsv220 activates Nrf2 to promote LvG6PDH, producing more NADPH and GSH to eliminate excessive ROS, thus escaping ROS‐induced host immune defense and facilitating successful WSSV infection. Therefore, our work elucidates the molecular mechanism underlying how viruses suppress host ROS.

The NADPH plays a key role in antioxidant synthesis and biosynthesis.^[^ ^42^ ^]^ The major production of intracellular NADPH occurs through the PPP, and the rate of NADPH generation is directly influenced by G6PDH as it serves as a rate‐limiting enzyme in the PPP.^[^ ^42^, ^56^ ^]^ High‐risk human papillomaviruses‐encoded E6E7 enhances NADPH levels and facilitates the proliferation of cervical cancer cells by activating PPP.^[^ ^44^ ^]^ The positive effect of G6PDH on Chilli veinal mottle virus infection is associated with ROS production regulated by respiratory burst oxidase homolog.^[^ ^57^ ^]^ Another study of the influenza virus demonstrates that the elevated level of G6PDH leads to NADPH overproduction and presents a positive association with virus replication.^[^ ^58^ ^]^ Previous studies have shown that the activity of LvG6PDH significantly increased after WSSV infection, mediating aerobic glycolysis.^[^ ^59^ ^]^ The Nrf2‐induced LvG6PDH activation would cause a metabolic shift into aerobic glycolysis, counteracting high ROS levels produced in response to viral infection and meeting the demand for macromolecule biosynthesis necessary for viral replication and assembly. Our studies and previous research substantiate the crucial role of G6PDH in regulating ROS dynamics and participating in aerobic glycolysis in response to viral infection, suggesting that metabolic reprogramming serves as a pivotal balancer between the ROS dynamics of the host and viral infection.

Interestingly, WSSV not only utilizes the Nrf2 pathway to regulate ROS levels but also exploits this pathway to support its replication. Specifically, WSSV constructs a positive feedback loop of wsv220‐Nrf2‐wsv051 to facilitate viral replication. This feedback loop continuously activates Nrf2 and theoretically maintains ROS stability during WSSV infection. ROS levels were elevated after 24 hpi, indicating that oxidative stress was induced again after the first WSSV replication cycle. However, WSSV genome replication continued to rise despite the ROS outbreak after 24 hpi. We attribute this to the continuous promotion of viral immediate early gene wsv051 expression by Nrf2 due to the positive feedback loop. Furthermore, wsv051 exhibits transcription factor activity and plays a critical role in WSSV infection and replication. And, the promoter activity of wsv051 is regulated by several host genes, including LvDorsal, LvSTAT, LvIKKβ, and LvIKKε.^[^ ^41^, ^62^, ^63^ ^]^ Our findings reveal the pivotal role of wsv051 in facilitating the expression of other viral genes. However, the regulatory mechanism of wsv051 on viral genes and its impact on host genes necessitates further investigation.

In summary, we reveal that WSSV exploits the Nrf2 pathway through its viral protein wsv220, allowing the virus to regulate ROS dynamics and establish a positive feedback loop that promotes viral replication. These results provide novel insights into how ROS regulation is manipulated during viral infections, expanding our understanding of the intricate interactions between viruses, hosts, and ROS.

## 4 Experimental Section

### Animals

Healthy shrimp *L. vannamei* (≈ 5 g) were acquired from the breeding base of the Ocean Building at the Zhuhai campus of Sun Yat‐Sen University, and the breeding base of Doumen Nanhai Institute of Oceanology. To avoid stress responses affecting the experiment, the shrimp were acclimated in a temporary tank at 27 °C for 1 week prior to the formal experiment. Sea salt was used to create artificial seawater (2.5 ‰). The shrimp were fed daily, and the water in the tanks was changed regularly to ensure the shrimp adapted to the experimental environment.

### Clone and Phylogenetic Analysis of Shrimp Nrf2

Partial fragments of LvNrf2 were obtained from the transcriptome database of *L. vannamei* in the lab. The cDNA of LvNrf2 was obtained by cloning from shrimp utilizing the SMARTer RACE 5′/3′ Kit (Takara 634 858). The sequence of LvNrf2 was exhibited in Figure S1 (Supporting Information). The information on all homologous Nrf2 proteins was obtained from NCBI. A neighbor‐joining (NJ) phylogenetic tree was created utilizing MEGA 6.0 software.

### Detection of ROS Levels

The ROS detection kit (Beyotime; cat. no. S0033S) was used to determine the ROS in hemocytes. Hemocytes were harvested from each treatment group (*n* = 5) and washed with PBS, then stained with 10 µm/L DCFH‐DA at room temperature for 20 min. Cells were mixed every 3–5 min. Then, cells were washed with PBS and tested utilizing a BD Accuri C6 Flow Cytometer (USA) with 488 nm excitation and 525 nm emission wavelengths.

### Detection of NADPH/NADP^+^ Ratios

The assay kit (Beyotime S0179) was used to quantify the NADPH/NADP^+^ levels in hemocytes (*n* = 30). Hemocytes were collected using a 1‐mL syringe and washed with PBS. Hemocytes were added to the extraction solution (200 µL) at 25 °C and treated for 10 min. Then mixture (100 µL) was heated at 60 °C to digest NADP^+^ and determine the amount of NADPH. Unheated samples were used to measure the total NADP^+^ and NADPH. The NADPH/NADP^+^ ratio was calculated using the formula: (NADPH) / (NADP_total_ – NADPH).

### Detection of GSH/GSSG Ratios

The GSH and GSSG assay kit (Beyotime S0053) was used to quantify the amounts of GSH and GSSG in shrimp hemocytes (*n* = 30). Hemocytes were harvested using a 1‐mL syringe. Hemocytes (50 µL) were mixed with protein removal reagent M (50 µL) and incubated on ice for 15 min. Then, the supernatant after centrifugation was used for total GSH determination. Another supernatant (100 µL) was treated with GSH removal auxiliary solution (20 µL) and GSH removal reagent working solution (4 µL) to eliminate GSH. The GSSG content was determined after incubating the reaction mixture at 25 °C for 1 h. GSH/GSSG ratio was calculated using the formula: (Total GSH – GSSG × 2) / GSSG.

### Real‐Time Quantitative PCR

Expression levels of LvNrf2, LvKeap1, LvGST1, LvGSH, LvG6PDH, LvEH, wsv220, IE1, and vp28 were measured utilizing the LightCycler 480 System (Roche, Basel, Germany). Each sample prepared a reaction volume (10 µL): including cDNA extracted from shrimp (50 ng), SYBR Green Pro Taq HS Premix (5 µL) (Agbio AG11701), and primers (20 µM) (Table S1, Supporting Information). The cycling program was performed as follows: 1 cycle of 95 °C for 30 s, followed by 40 cycles of 95 °C for 5 s, 60 °C for 30s. Genes transcription levels were calculated utilizing the Livak (2^−ΔΔCT^) method.

### Western Blotting Assay

The protein samples used in this study were separated using SDS‐PAGE gels. Then, the same was transferred to PVDF membranes (Merck IPVH00010) and incubated with the corresponding antibodies. Primary antibodies used in this study consisted of rabbit anti‐GFP (Sigma G1544‐100UL), rabbit anti‐HA (Sigma H6908‐100UL), and rabbit anti‐FLAG (Sigma F7425). The secondary antibodies used in this study consisted of anti‐mouse IgG HRP‐conjugate (Promega W4021) and anti‐rabbit IgG HRP‐conjugate (Promega W4011). These antibodies were diluted in TBS‐T containing BSA (0.5%) for 2 h. The density of the immunoblotted protein bands was quantified using ImageJ software 1.6.0.

### Dual‐Luciferase Reporter Assay

To assess the impact of LvNrf2 or wsv220 on LvNrf2 target gene promoters, *Drosophila* S2 cells were seeded in a 24‐well plate at an appropriate suitable density. Each group added firefly luciferase reporter‐gene plasmids of LvNrf2 target genes (0.7 µg), expression plasmids of LvNrf2 or wsv220 (0.7 µg), pRL‐TK renilla luciferase plasmid (0.1 µg), and FugENE transfection reagent (1.8 µL) (Promega E2311), followed by cell harvesting, lysis at 48 h post‐transfection of the lysate (60%) was measured reporter gene induction via the Dual‐Glo Luciferase Assay System (Promega E2920). The rest of the S2 cell lysate (40%) was utilized for quantifying protein expression levels.

### RNAi Assay

The T7 Ribo^MAX^ Express RNAi System kit (Promega P1700) was utilized for the generation of dsLvNrf2, dsLvKeap1, dswsv220, and dseGFP using specific primers (Table S1, Supporting Information). DsRNA (100 ng µL^−1^) was subjected to gel electrophoresis for quality assessment and then diluted in PBS. Experimental animals were administered an intraperitoneal injection of dsRNAs (2 µg g^−1^ shrimp, *n* = 30). Hemocytes were harvested at 48 h post‐dsRNA injection to employ total RNA extraction and assessed by qPCR to evaluate RNAi efficiency.

### Shrimp Mortality and Detection of Viral Loads

The 100 g muscle of WSSV‐infected shrimp was homogenized into 1 mL PBS. This viral crude extract was diluted into PBS (≈1 × 10^5^ copies in 50 µL) according to the previously reported. Shrimp were intraperitoneally injected with WSSV (1 × 10^5^ copies). The infected shrimp were cultured for a week. The mortality was recorded every 8 hours for survival rate analysis. To quantify viral loads of WSSV in infected shrimp, absolute quantitative PCR was performed using primers of wsv069 (Table S1, Supporting Information). The DNA amplicon of wsv069 from the WSSV genome (AF332093.2) was subcloned into the pMD19‐T plasmid to generate a standard curve for absolute qPCR. The genomic DNA was extracted from muscle tissue utilizing the Marine Animal Tissue Genomic DNA Extraction Kit (TianGen Biochemical Technology DP324). Then, the extracted DNA samples (*n* = 10) were subjected to absolute qPCR. Each sample from one shrimp was analyzed in triplicate. Viral loads of WSSV were calculated according to the standard curve and normalized to 0.1 µg shrimp tissue DNA.

### Co‐IP Assay

Co‐IP assays were conducted to validate protein interactions. *Drosophila* S2 cells transferred with pAC5.1A‐LvNrf2, pAC5.1A‐LvKeap1, pAC5.1A‐wsv220, and its mutant were collected after 48 h, then added IP lysis buffer. The supernatant obtained after lysis was mixed with agarose affinity gel containing anti‐Flag (Sigma A2220) or anti‐HA (Sigma A2095) at 4 °C for 4 h. Then, agarose gels were washed with PBS and subjected to western blotting assay. Total cell lysate (5 %) was examined as a control.

### Intracellular Competitive Combination Experiment

To assess the impact of wsv220 on the interaction between LvNrf2 and LvKeap1 in *Drosophila* S2 cells, cells transfected with pAC5.1A‐LvKeap1‐HA (10 µg), pAC5.1A‐LvNrf2‐Flag (10 µg), and varying amounts of pAC5.1A‐wsv220‐Flag (0, 2.5, 5, 7.5, 10, and 30 µg). In each group, the plasmid pAc5.1 was used to ensure a total transfection capacity of 50 µg. After 48 h of transfection, cells were collected for Co‐IP as above described.

### Recombinant Protein Purification and Injection In Vivo

The ORF of wsv220 was genetically modified by incorporating a cell‐penetrating TAT peptide (TATGGCAGGAAGAAGCGGAGACAGCGACGAAGA) and then cloned into the pGEX‐4T‐1, resulting in the production of a recombinant protein (rTAT‐wsv220‐GST) expressed in *Escherichia coli* (*E. coli*) strain BL21. The bacteria transformed with rTAT‐wsv220‐GST were cultured in Luria–Bertani medium (Solarbio L1015) supplemented with 500 µg mL^−1^ of ampicillin (Macklin A830931) at 37 °C until OD600 reached 0.8. Then, 1 mm isopropyl‐β‐D‐thiogalactopyranoside (IPTG, Promega V3955) was used to induce the expression of wsv220 at 37 °C for 6 h. Proteins were purified using a Pierce^TM^ GST protein interaction pull‐down kit (ThermoFisher 21 516). The pET‐B2M‐rTAT‐LvNrf2, pET‐32a‐rTAT‐LvKelch, or pET‐32a‐rTAT plasmid was transfected into *E. coli* strain BL21 and cultured in Luria–Bertani medium supplemented with 500 µg mL^−1^ ampicillin at 37 °C for 3 h, followed by induction with 0.1 mm IPTG at 12 °C for 12 h. Proteins were purified using HisSep Ni‐NTA Agarose Resin (Yesen 20502ES50). The purified proteins were validated by SDS‐PAGE, followed by coomassie blue staining (Figure S6A,B,G, Supporting Information). For the overexpression experiment in vivo, each shrimp was injected with 10 µg of TAT‐tagged recombinant protein. Hemocytes treated with TAT‐tag recombinant proteins were collected at 12 h post‐injection to obtain protein samples for western blotting (*n* = 30).

### Electrophoretic Mobility Shift Assay

The ARE motifs and mutated ARE motifs were designed as either biotinylated or unbiotinylated oligonucleotides (Table S1, Supporting Information). Oligonucleotides (10 µm) were annealed to form double‐stranded probes. Recombinant protein was prepared from *E. coli* strain BL21 transfected with pMal‐c2X‐LvNrf2 or pMal‐c2X plasmid, and cultured in Luria–Bertani medium supplemented with 500 µg mL^−1^ ampicillin at 37 °C for 5 h, followed by induction with IPTG (0.05 mm) at 37 °C for 5 h. Proteins were purified using the PurKine MBP‐Tag Protein Purification Kit (Abbkine KTP2020) and confirmed by SDS‐PAGE followed by coomassie blue staining (Figure S7C,D, Supporting Information). For an incubation system (20 µL), sample was supplemented with unmutated‐bio‐probes (20 fmol) and purified LvNrf2‐MBP or MBP (2 µg). In competition binding assays, complexes of wild‐type probes and proteins were challenged with unbiotinylated probes (or M‐unbio probes) at tenfold, 50‐fold, or 100‐fold molar excess compared to labeled probes. The EMSA was performed following the protocols provided by the LightShift Chemiluminescent EMSA Kit (Thermo Fisher 20 148).

### Chromatin Immunoprecipitation Assay

ChIP assays were conducted using the EZ‐ChIP kit (Merck millipore 17–371). Gills were collected from infected shrimp at 48 hpi and lysed with ChIP cell lysis buffer and SDS lysis buffer for the extraction of nuclear proteins. The supernatants, obtained after ultrasonication followed by centrifugation, were incubated at 4 °C for 12 h with anti‐LvNrf2 antibody (Genecreate antibody customization), and then, mixed with protein G magnetic beads. The protein‐DNA complex was subjected to thermal reversal of cross‐links by being treated at 62 °C for 2 h, then heated at 95 °C for 10 min. The obtained DNA was processed with an E.Z.N.A.^®^ cycle pure kit (Omega D6492) and subjected to semi‐quantitative RT‐PCR analysis using specificity primers (Table S1, Supporting Information).

### Semi‐Quantitative RT‐PCR

Genomic DNA was extracted from hemocytes at 48 h post‐WSSV infection to detect the expression of wsv051, wsv069, and wsv187, vp28, vp26, and vp15. Specific primers are shown in Table S1 (Supporting Information), and β‐actin was amplified as a control. Each group prepared nine PCR reactions. Beginning from the 22nd cycle, one tube was removed every two cycles until all cycles were completed. The cycle number of the PCR plateau phase was determined by agarose gel electrophoresis. Subsequently, PCR was carried out under specified conditions. The variation in expression levels of viral genes within each group was assessed by examining band intensity in the agarose gel. The semi‐quantitative PCR process was conducted as follows: 1 cycle at 94 °C for 3 min, 26 cycles at 94 °C for 30 s, 55 °C for 45 s, and 72 °C for 30 s, followed by 1 cycle at 72 °C for 10 min.

### Statistics Analysis

All data are presented as mean values ± SD (standard deviation) of at least three independent experiments. Statistical values were calculated using a two‐tailed Student's *t‐test*. Asterisks indicate statistical significance: ns means no significance; ^*^ means *p* < 0.05; ^**^ means *p* < 0.01.

### Ethical Statement

This study does not contain any studies with human participants and endangered or protected species. No specific permits were required for the described field studies. No specific permissions were required for access to the artificial aquarium in Zhuhai and Maoming, Guangdong Province, China.

## Conflict of Interest

The authors declare no conflict of interest.

## Author Contributions

Y.H.C. and J.G.H. conceived the presented idea and designed the experiments. H.H.H. and K.Y. conducted the experiments and analyzed the data. J.M.P. conducted the protein purification and western blotting assay. J.G.H., Y.H.C., C.Z.L., and S.P.W. supervised the study. H.H.H. wrote the manuscript and generated the figures. C.Z.L., Y.H.C., and J.G.H reviewed and edited the manuscript. All authors discussed the results, commented on, and proofread the manuscript. The principal investigator is J.G.H.

## Supporting information



Supporting Information

## Data Availability

The data that support the findings of this study are available from the corresponding author upon reasonable request.
