# Supplementary material for: Shrimp Virus Regulates ROS Dynamics via the Nrf2 Pathway to Facilitate Viral Replication
Source: Adv Sci (Weinh). 2025 Mar 16;12(18):2407695. doi: 10.1002/advs.202407695 (PMC12079347; doi:10.1002/advs.202407695)
Supplement: Supplementary file 1 — Supporting Information [file ADVS-12-2407695-s001.docx]

Supporting Information

**Shrimp Virus Regulates ROS Dynamics via the Nrf2 Pathway to Facilitate Viral Replication**

*Honghui He,* *Kai Yuan, Junming Pan, Shaoping Weng, Chaozheng Li*,* *Yihong Chen***, and* *Jianguo He**

This file contains the Supplementary Figures 1 to 13 and Table 1 to 3.


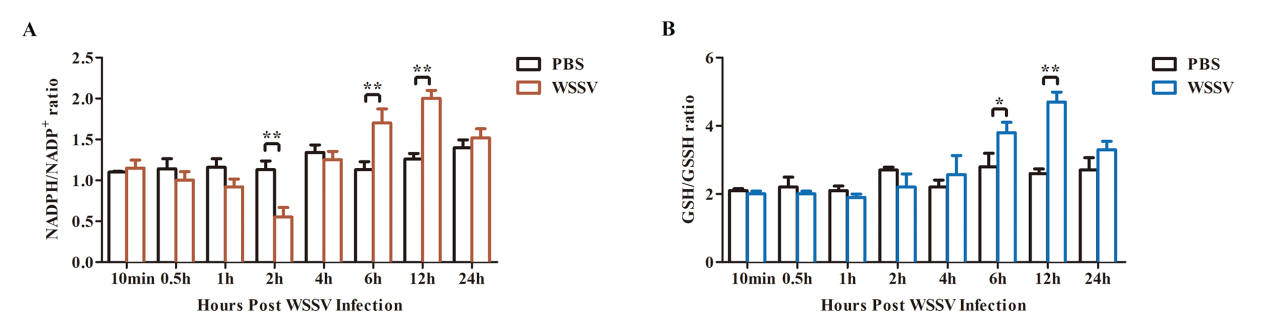


**Figure S1. The dynamic changes of the** **ratios of (A) NADPH/NADP^+^ and (B) GSH/GSSH in hemocytes following WSSV infection.**


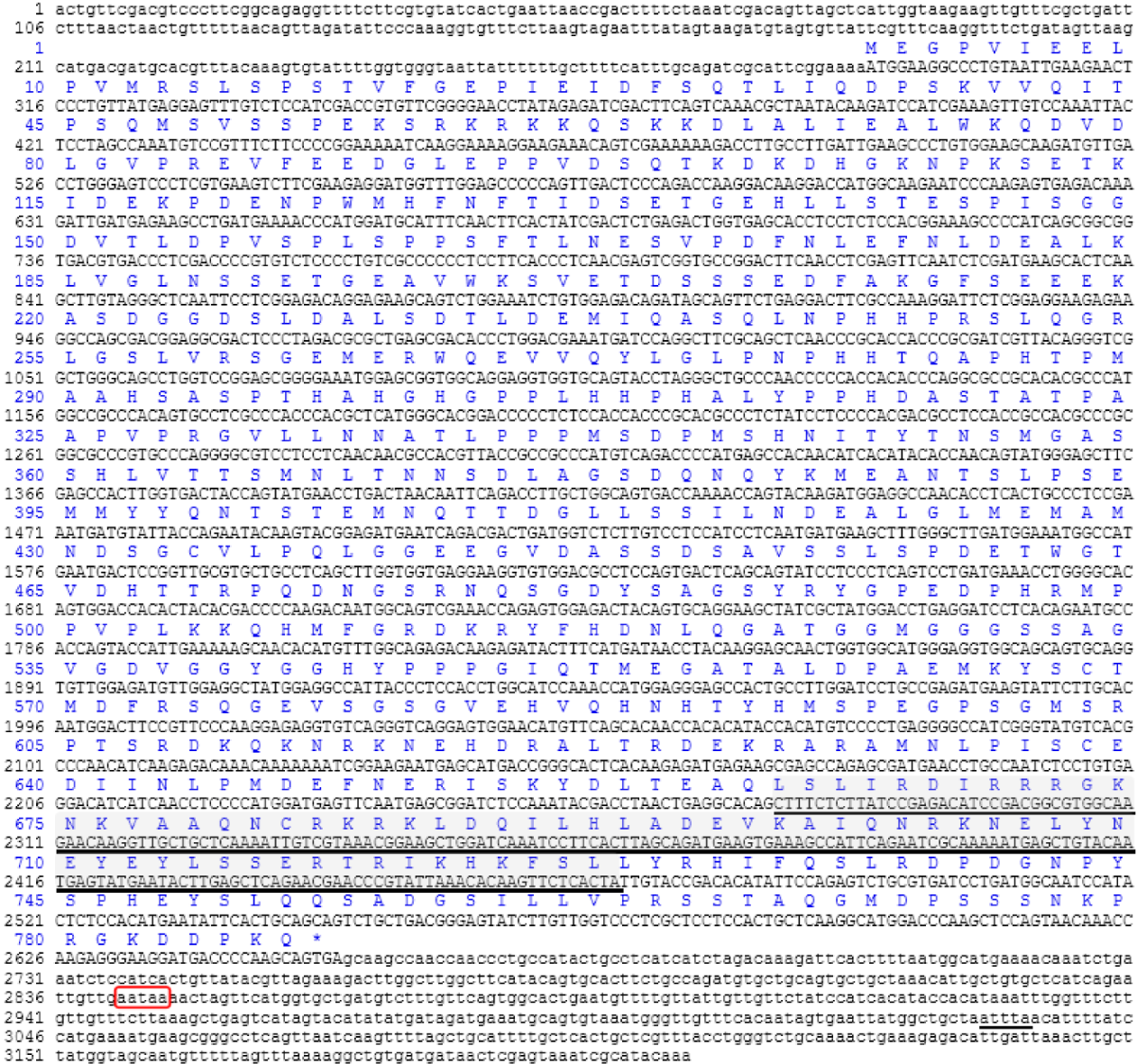


**Figure S2. Nucleotide and deduced amino acid sequence of LvNrf2.**

The ORF of LvNrf2 was shown in uppercase letters; the 5′- and 3′-UTRs are shown in lowercase letters. Nucleotides and amino acids are numbered on the left of the sequences. The conserved domains are shaded. The poly (A) signals (aataaa) are boxed. The 3′-UTR instability motifs (attta) are underlined.


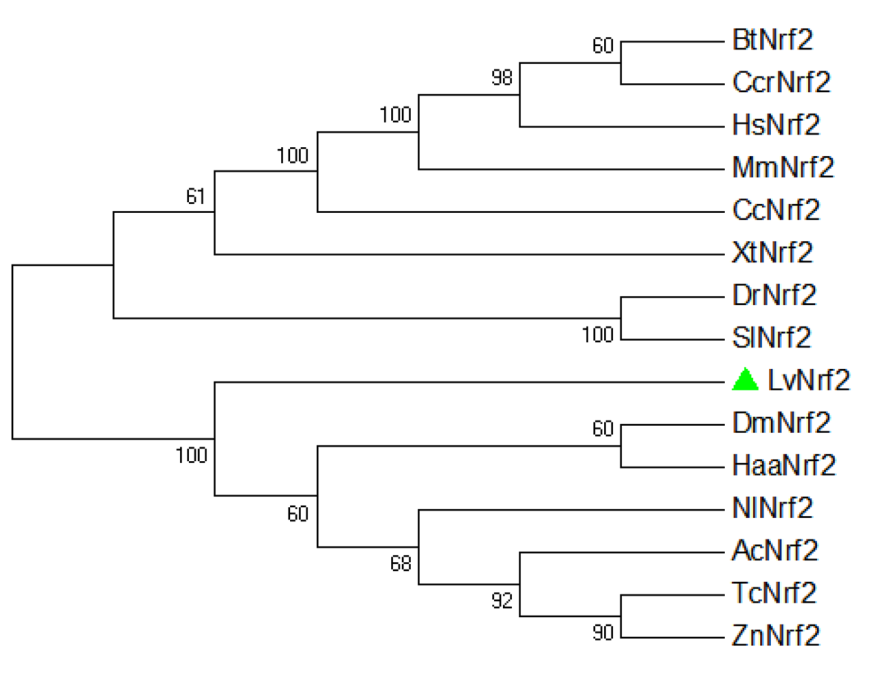


**Figure S3. Phylogenetic tree of LvNrf2.**

The tree was constructed with the neighbour-joining (NJ) method based on the alignment of 15 Nrf2 full-length protein sequences by utilizing MEGA 5.0 software. The bootstrap values of 1000 replicates (%) were indicated on the branch nodes. LvNrf2 was indicated in green triangles.

**
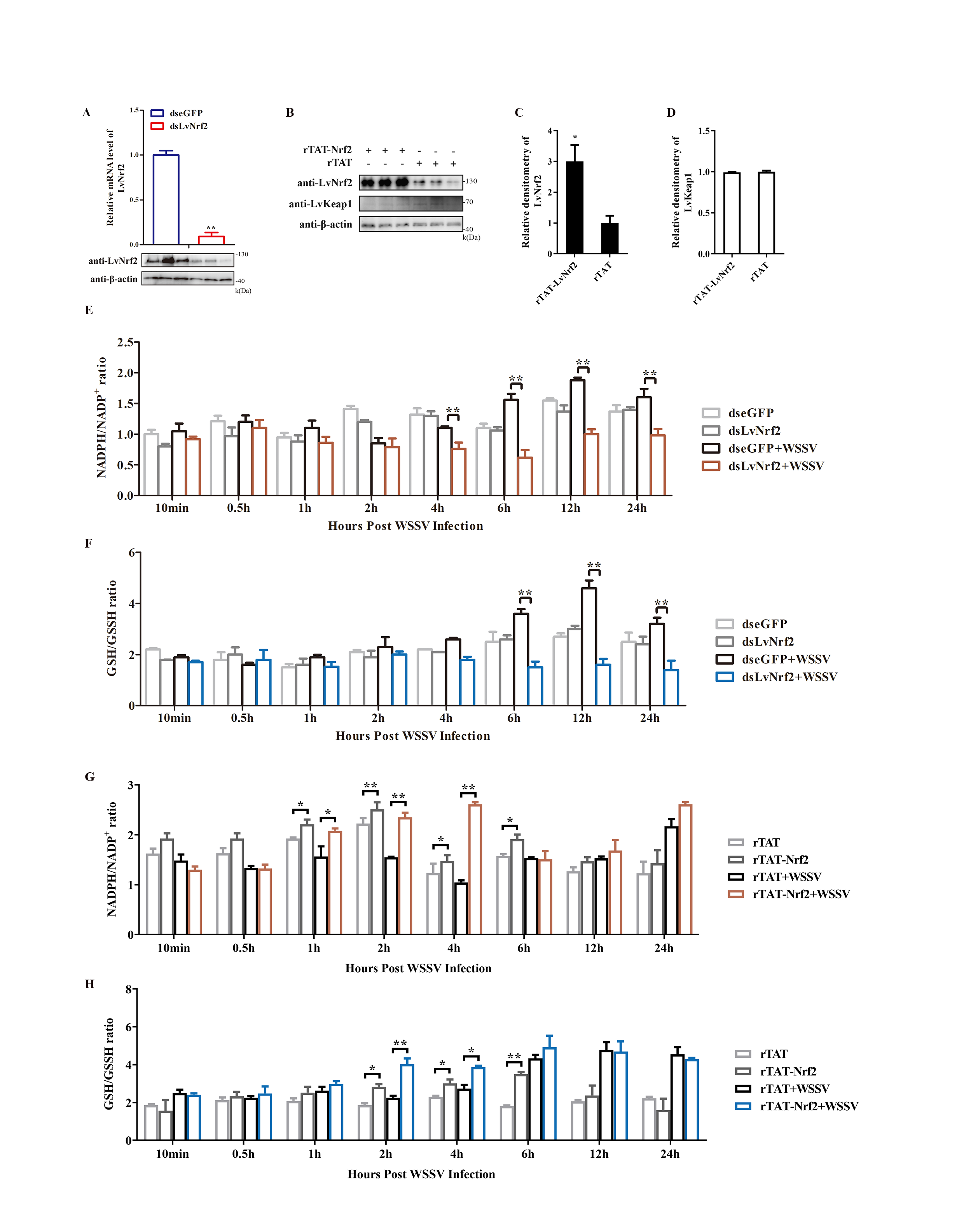
Figure S4. LvNrf2 elevated levels of NADPH and GSH to restore the cellular redox balance during WSSV infection**. (A) Silencing efficiencies of LvNrf2 in hemocytes were assessed by qPCR and western blotting at 48 hours post-dsRNA injection. (B) Expression levels of LvNrf2 and LvKeap1 in hemocytes was detected by western blotting in rTAT- or rTAT-LvNrf2- injected shrimp post-WSSV infection. (C-D) Statistical analysis of LvNrf2 (C) and LvKeap1 (D) by WCIF ImageJ software corresponding to (B). (E-F) The ratios of (E) NADPH/NADP^+^ and (F) GSH/GSSH in dseGFP- or dsLvNrf2- injected shrimp post-WSSV infection. (G-H) The ratios of (G) NADPH/NADP^+^ and (H) GSH/GSSH in rTAT- or rTAT-LvNrf2- injected shrimp post-WSSV infection.


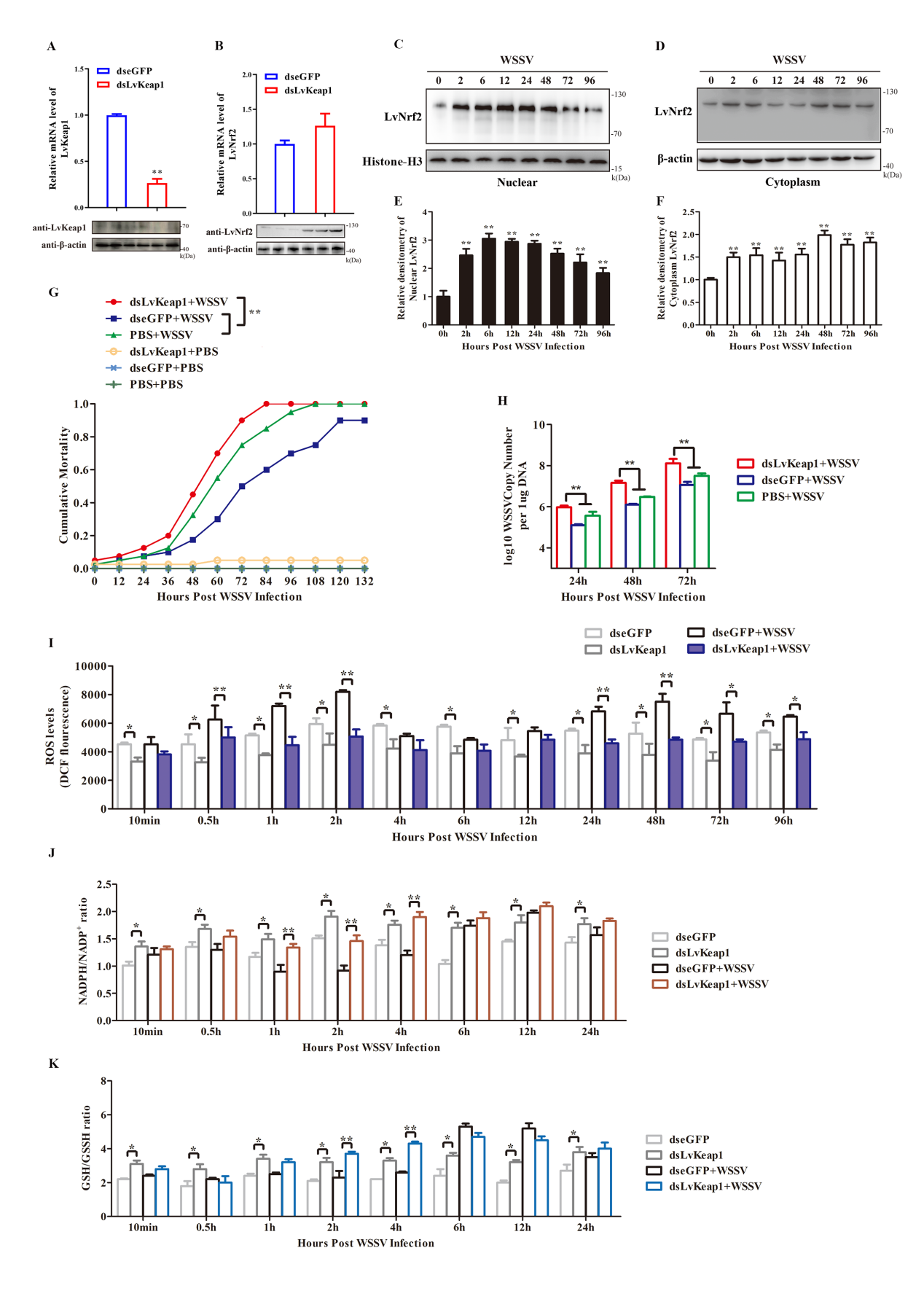


**Figure S5. LvKeap1 knockdown activated LvNrf2 to facilitate WSSV infection**. (A-B) Silencing efficiencies of LvKeap1 and LvNrf2 in hemocytes were assessed by qPCR and western blotting at 48 hours post-dsRNA injection. (C-D) Expression levels of LvNrf2 in nucleus (C) and cytoplasm (D) were detected by western blotting in dsKeap1-injected shrimp post-WSSV infection. (E-F) Statistical analysis of LvNrf2 in the nucleus (E) and cytoplasm (F) by WCIF ImageJ software corresponding to (C-D). (G) LvKeap1 knockdown decreases the resistance of shrimp to WSSV infection. (H) LvKeap1 knockdown results in increased WSSV replication levels in muscle tissue. (I) ROS levels in hemocytes of dsLvKeap1-injected shrimp after WSSV or PBS injection. (J-K) The ratios of (I) NADPH/NADP^+^ and (J) GSH/GSSH in dseGFP- or dsLvKeap1- injected shrimp post-WSSV infection.


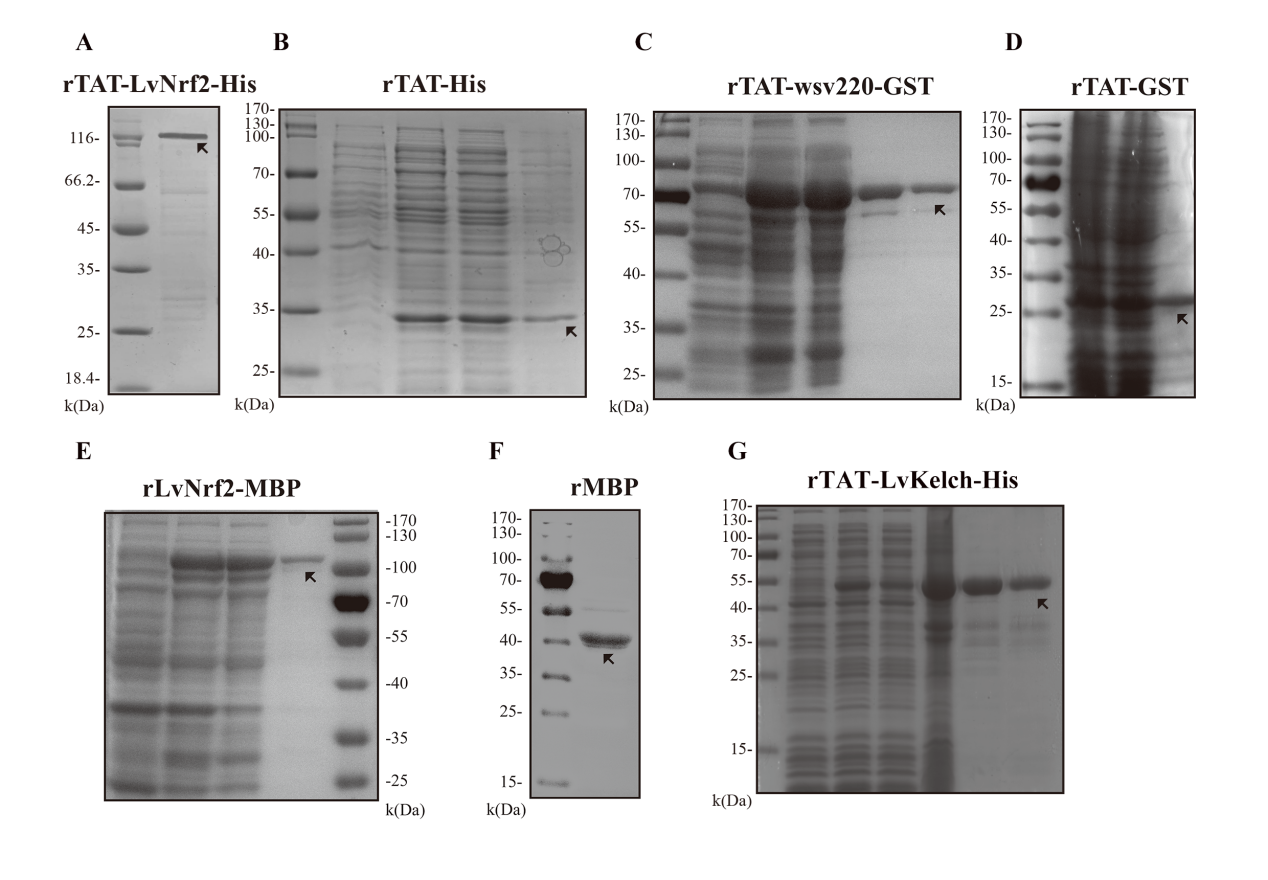


**Figure S6. Recombinant protein purification** **related to Fig 2F-H, 4D, 8C and 9.** (A-B) rTAT-LvNrf2-His (A) and rTAT-His (B) were used for *in vivo* protein injection experiment in Fig. 2F-H. The ORF of LvNrf2 was cloned into the modified pET-B2M-rTAT for the recombinant proteins entering the shrimp cells. (C-D) rTAT-wsv220-GST (C) and rTAT-GST (D) were used for *in vivo* protein injection experiment in Fig. 4C. The ORF of wsv220 was cloned into the modified pGEX-rTAT for the recombinant proteins entering the shrimp cells. (E-F) rMBP-LvNrf2 (E) and rMBP (F) were used for EMSA experiment in Fig. 8C. The ORF of LvNrf2 was cloned into **pMal-c2X** to obtain LvNrf2-MBP recombinant protein. These four purification proteins were analyzed using SDS-PAGE and stained with Coomassie blue. (G) rTAT-LvKelch-His was used for *in vivo* protein injection experiment in Fig. 9. The ORF of LvKeap1 was cloned into the modified pET-B2M-rTAT for the recombinant proteins entering the shrimp cells.


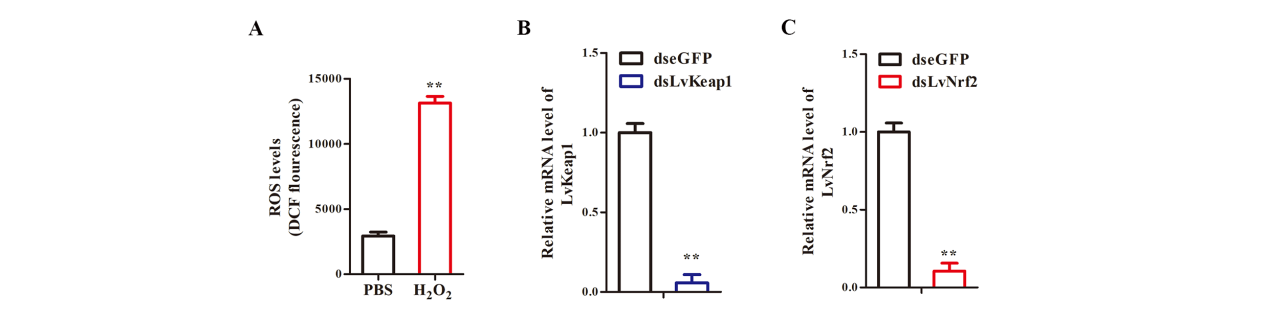


**Figure S7. H_2_O_2_ induced oxidative stress *in vivo*.** (A) ROS levels in hemocytes stimulated by 50 μM H_2_O_2_ for 48 hours. (B-C) Silencing efficiencies (B) of LvNrf2 and LvKeap1 (C) in hemocytes at 48 hours post-dsRNA injection, assessed by qRT-PCR.


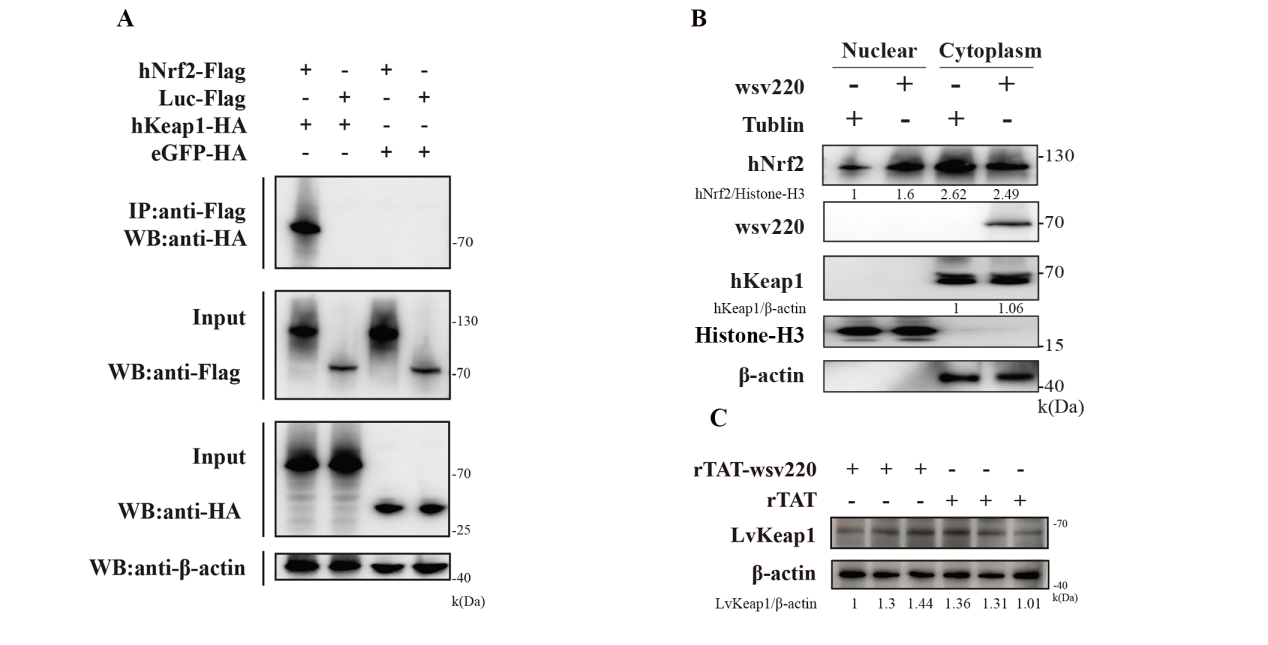


**Figure S8. wsv220 activated LvNrf2.** (A) Co-IP analysis of the interaction between hNrf2 and hKeap1. (B) Western blot analysis shows that overexpression of wsv220 in 293T cells promotes LvNrf2 nuclear translocation, while did not affect the expression of LvKeap1. (C) Expression level of LvKeap1 was detected by western blotting in rTAT-wsv220- and rTAT-injected shrimp.


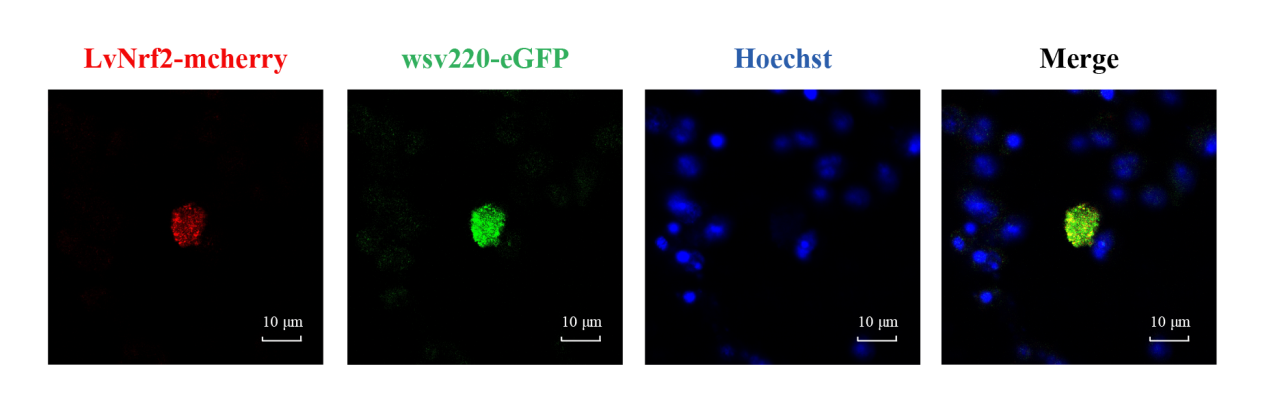


**Figure S9. Immunofluorescence assay of co-expression of LvNrf2 and wsv220** **in *Drosophila* S2 cells.** Red fluorescence represents LvNrf2, green fluorescence represents wsv220, and nuclei were stained with DAPI (blue).


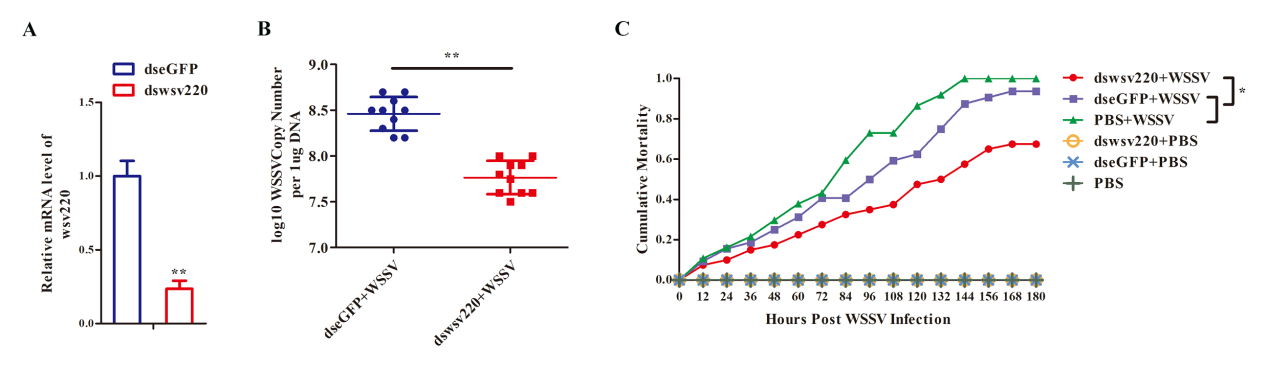


**Figure S10.** **Knockdown of wsv220 reduced the pathogenicity of WSSV.** (A) Silencing efficiencies of wsv220 in hemocytes were assessed by qRT-PCR at 48 hours post-dsRNA injection. (B) Knockdown of wsv220 depressed viral loads in muscle at 48 hours post-dsRNA injection. WSSV was inoculated simultaneously with dswsv220. (C) Knockdown of wsv220 decreased mortality post-WSSV infection. The death of shrimp was recorded every 8 hours for cumulative mortality.


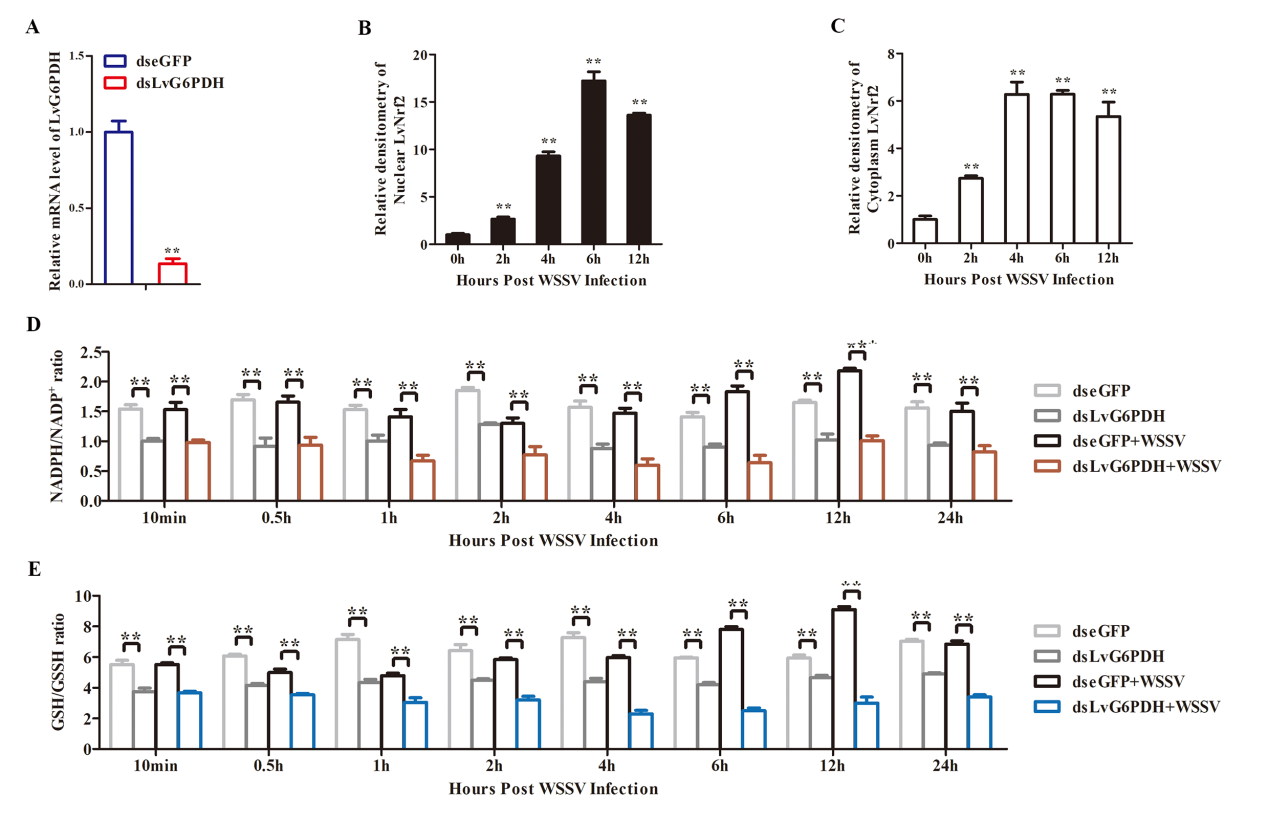


**Figure S11. Knockdown of LvG6PDH reduced the ratios of NADPH/NADP^+^ and GSH/GSSH in hemocytes following WSSV infection.** (A) Silencing efficiencies of LvG6PDH in hemocytes were assessed by qRT-PCR at 48 hours post-dsRNA infection. (B-C) Statistical analysis of LvNrf2 in the nucleus (B) and cytoplasm (C) by WCIF ImageJ software corresponding to Fig 7B. (D-E) The ratios of (D) NADPH/NADP^+^ and (E) GSH/GSSH in dseGFP- or dsLvG6PDH- injected shrimp post-WSSV infection.


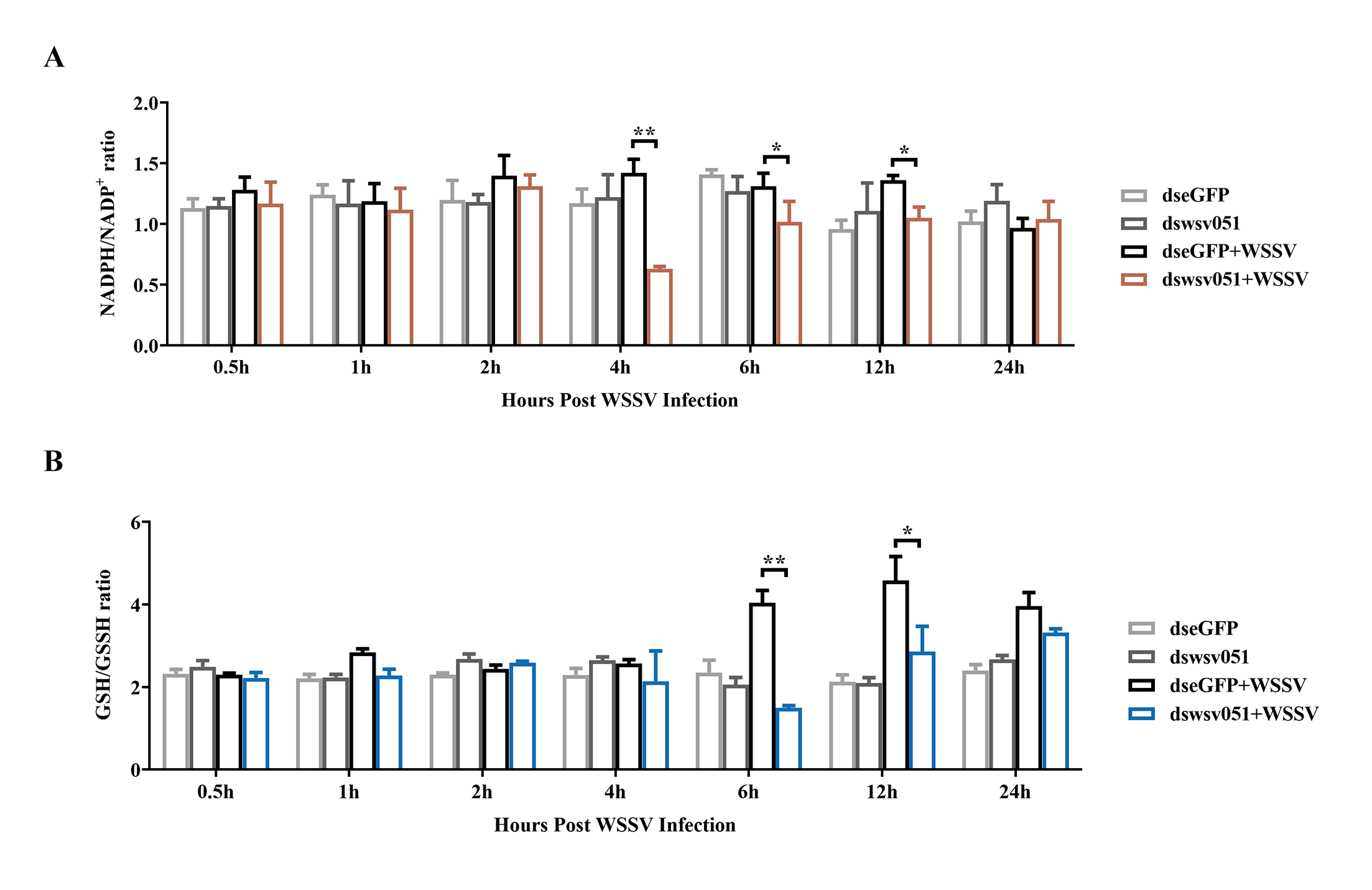
**Figure S12. Knockdown of wsv051 reduced the ratios of NADPH/NADP^+^ and GSH/GSSH in hemocytes following WSSV infection.** (A-B) The ratios of (A) NADPH/NADP^+^ and (B) GSH/GSSH in dseGFP- or dswsv051- injected shrimp post-WSSV infection.


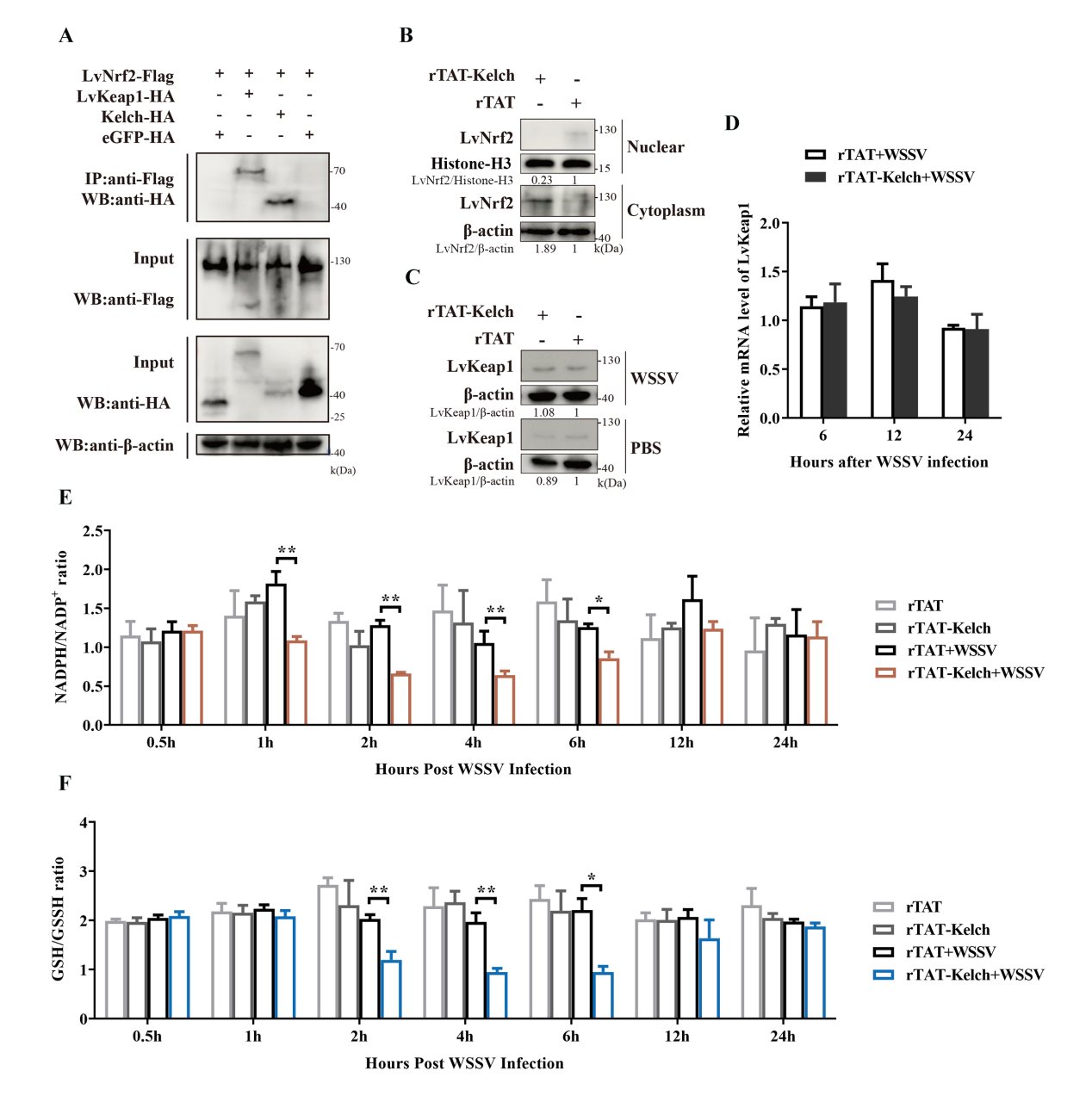


**Figure S13. rTAT-Kelch-injection activated LvNrf2 to reduce the ratios of NADPH/NADP^+^ and GSH/GSSH in hemocytes following WSSV infection.** (A) Co-IP analysis of the interaction between LvNrf2 and Kelch in *Drosophila* S2 cells. (B) Expression levels of LvNrf2 in nucleus and cytoplasm were detected by western blotting in rTAT- or rTAT-Kelch- injected shrimp post-WSSV infection. (C) Expression levels of LvKeap1 were detected by western blotting in rTAT- or rTAT-Kelch-injected shrimp. (D-E) The ratios of (D) NADPH/NADP^+^ and (E) GSH/GSSH in rTAT- or rTAT-Kelch- injected shrimp post-WSSV infection.

**Table S1. Summary of primers used in this study.**

| **Names** | **Sequences (5'–3')** |
| --- | --- |
| **qPCR assay** |  |
| EF-1α-F | GTATTGGAACAGTGCCCGTG |
| EF-1α-R | ACCAGGGACAGCCTCAGTAAG |
| LvNrf2-F | CCAAACCATGGAGGGAGCCA |
| LvNrf2-R | CCACTCCTGACCCTGACACC |
| LvKeap1-F | CGCCACGCAACAGATTAGGG |
| LvKeap1-R | TCCGTGCCTTTGACATGGGT |
| LvG6PDH-F | CGCCTCGGTCTTCATCTCGT |
| LvG6PDH-R | GCAGGTGGTTCTGCATGACG |
| LvGSH-F | CGACCCAAGCCAGCTCTTCT |
| LvGSH-R | GGGTCGAGTATCCGCGTCAT |
| LvGST1-F | CTCGGCCTCGACTTTCCCAA |
| LvGST1-R | GTGCTGGCGAGCAATGTGAC |
| LvEH-F | GGCGAAACCAGTTGCAGGAG |
| LvEH-R | CGGGTTTATCGGAGGAGCCA |
| wsv220-F | AAGCGAGAGAGTGACGCCAT |
| wsv220-R | GGCCGGTGCTTTACAACAGG |
| wsv051-F | CCACAGAGGTCCCAAGTGTGT |
| wsv051-R | GACCCAGGGCCTTCTTCTCC |
| VP28-F | AACACCTCCTCCTTCACCC |
| VP28-R | GGTCTCAGTGCCAGAGTAGGT |
| WSSV32678-F | TGTTTTCTGTATGTAATGCGTGTAGGT |
| WSSV32753-R | CCCACTCCATGGCCTTCA |
| TaqMan probe WSSV32706 | CAAGTACCCAGGCCCAGTGTCATACGTT |
| **Dual-luciferase reporter assay** |  |
| LvG6PDH-F | CGGGGTACCATGTCACGCCCGGTCGTAA |
| LvG6PDH-R | CCGCTCGAGTTACAAGTGACTGGGTTGTGTC |
| LvGSH-F | CGGGGTACCATGAATGTGCTCCACAACTCC |
| LvGSH-R | CCGCTCGAGCTAAAACTCCTGCTGGAGG |
| LvGST1-F | CGGGGTACCATGGCGCCCGTGTTGGCATA |
| LvGST1-R | TCCCCCCGGGCTATTTATTTCCAAACTTGGCCATCTTG |
| LvEH-F | CGGGGTACCATGGGTATCATCCGGAAGG |
| LvEH-R | CCGCTCGAGCTAAAATGCTGATATCCATTCATCAGAG |
| LvNrf2-F | CGGGGTACCATGGAAGGCCCTGTAATTGAAG |
| LvNrf2-R | TGCTCTAGACTGCTTGGGGTCATCCTTC |
| LvKeap1-F | ATAAGAATGCGGCCGCATGGCTGGTACCCAG |
| LvKeap1-R | TGCTCTAGACTATCTCGCATCAGCA |
| wsv220-F | CGGGGTACCATGGCAGGGAATAGAACCCA |
| wsv220-R | TGCTCTAGATTATACGGGAAAATTCTCCAGAAGG |
| eGFP-F | CGGGGTACCATGGTGAGCAAGGGCGAGGA |
| eGFP-R | TGCTCTAGACTTGTACAGCTCGTCCATGC |
| **dsRNA synthesis** |  |
| LvNrf2-T7-F | TAATACGACTCACTATAGGGCTCAATTCCTCGGAGACAG |
| LvNrf2-R | ACTGCCAGCAAGGTCTGAAT |
| LvNrf2-F | GCTCAATTCCTCGGAGACAG |
| LvNrf2-T7-R | TAATACGACTCACTATAGGACTGCCAGCAAGGTCTGAAT |
| LvKeap1-T7-F | TAATACGACTCACTATAGGCCACGATTCTTGACAGAGCA |
| LvKeap1-R | GTCCAGTACTGCCACCCCTA |
| LvKeap1-F | CCACGATTCTTGACAGAGCA |
| LvKeap1-T7-R | TAATACGACTCACTATAGGGTCCAGTACTGCCACCCCTA |
| wsv220-T7-F | TAATACGACTCACTATAGGGGAGAGTTTTCCTCCCGTTC |
| wsv220-R | CGGATAAAGGATGTCGAGGA |
| wsv220-F | GGAGAGTTTTCCTCCCGTTC |
| wsv220-T7-R | TAATACGACTCACTATAGGCGGATAAAGGATGTCGAGGA |
| LvG6PDH-T7-F | TAATACGACTCACTATAGGCGAGAAGGTGAAGGTCCTGA |
| LvG6PDH-R | CCAGCTCATCACTCCTGACA |
| LvG6PDH-F | CGAGAAGGTGAAGGTCCTGA |
| LvG6PDH-T7-R | TAATACGACTCACTATAGGCCAGCTCATCACTCCTGACA |
| wsv051-T7-F | TAATACGACTCACTATAGGCGTATGGAGACGGAATTGGT |
| wsv051-R | TTTTTATGAGGTTGGCCCTG |
| wsv051-F | CGTATGGAGACGGAATTGGT |
| wsv051-T7-R | TAATACGACTCACTATAGGTTTTTATGAGGTTGGCCCTG |
| LvKelch-T7-F | TAATACGACTCACTATAGGGATGACAACACTTGGGCCTT |
| LvKelch-R | CAGATCTGCCCCAAGGTAAA |
| LvKelch-F | GATGACAACACTTGGGCCTT |
| LvKelch-T7-R | TAATACGACTCACTATAGGCAGATCTGCCCCAAGGTAAA |
| eGFP-T7-F | TAATACGACTCACTATAGGACGTAAACGGCCACAAGTTC |
| eGFP-R | TGTTCTGCTGGTAGTGGTCG |
| eGFP-F | ACGTAAACGGCCACAAGTTC |
| eGFP-T7-R | TAATACGACTCACTATAGGTGTTCTGCTGGTAGTGGTCG |
| **ChIP assay** |  |
| ChIP-wsv051-F | ACCAGTTCCAGGAAGAATGC |
| ChIP-wsv051-R | CGTCGTCTTCGTCAGAGAC |
| **Semiquantitative RT-PCR** |  |
| EF-1α-F | TCGCTTCAAGGAAATCCACAAG |
| EF-1α-R | AAGGTCTCCACGCACATAGGC |
| wsv051-F | ATCGTTGGCCACCATGGAC |
| wsv051-R | TAGTAGCTAAAGTTGTTGCGACG |
| wsv069-F | CGTACGGCAAGGAAAGTGTG |
| wsv069-R | ATGTCAAATCAGAATGACCCACTC |
| wsv187-F | GATACAAAACGGTTGGGGTTTTC |
| wsv187-R | ATATGGCCACTTCTCACACC |
| vp28-F | TCACAACACTGTGACCAAGAC |
| vp28-R | CGTGCACGTACATGTCGAAAA |
| vp26-F | CGTCGTCGCTAATTATGATCAG |
| vp26-R | CGATCACATTCTTGGAGGTG |
| vp15-F | ATGGTTGCCCGAAGCTCC |
| vp15-R | TTAACGCCTTGACTTGCGGG |
| **EMSA assay** |  |
| Probe-wsv051-WT-F | CCGAGGTTCGGCAGTCAGTTCCAGA |
| Probe-wsv051-WT-R | TCTGGAACTGACTGCCGAACCTCGG |
| Probe-wsv051-MUT-F | CCGAGGTTTAATGACTGGTTCCAGA |
| Probe-wsv051-MUT-R | TCTGGAACCAGTCATTAAACCTCGG |

**Table S2.** **The data of the ratios of NADPH/NADP^+^ ralated to Figure S1A**

|  |  | 10min | 0.5h | 1h | 2h | 4h | 6h | 12h | 24h |
| --- | --- | --- | --- | --- | --- | --- | --- | --- | --- |
| NADP_total_ (μM) | PBS | 1.735 | 2.945 | 2.431 | 1.074 | 2.199 | 1.718 | 2.508 | 1.446 |
|  |  | 2.079 | 2.210 | 1.680 | 1.106 | 1.527 | 1.973 | 2.364 | 1.975 |
|  |  | 1.785 | 3.036 | 2.748 | 2.934 | 2.110 | 2.486 | 2.619 | 1.544 |
|  | WSSV | 1.910 | 1.443 | 2.137 | 3.088 | 2.368 | 1.775 | 1.901 | 0.770 |
|  |  | 1.775 | 2.258 | 1.797 | 3.379 | 2.479 | 1.367 | 1.979 | 0.764 |
|  |  | 1.741 | 2.229 | 2.047 | 2.054 | 1.769 | 1.535 | 1.889 | 0.890 |
| NADPH (μM) | PBS | 0.930 | 1.520 | 1.303 | 0.589 | 1.285 | 0.957 | 1.357 | 0.856 |
|  |  | 1.113 | 1.257 | 0.857 | 0.606 | 0.895 | 0.996 | 1.357 | 1.157 |
|  |  | 0.948 | 1.556 | 1.551 | 1.457 | 1.157 | 1.316 | 1.458 | 0.957 |
|  | WSSV | 1.038 | 0.756 | 0.958 | 0.957 | 1.347 | 1.056 | 1.258 | 0.467 |
|  |  | 0.895 | 1.157 | 0.857 | 1.105 | 1.416 | 0.885 | 1.357 | 0.476 |
|  |  | 0.966 | 1.258 | 1.047 | 0.857 | 0.929 | 0.994 | 1.258 | 0.517 |
| NADP^+^ (μM) | PBS | 0.805 | 1.426 | 1.127 | 0.484 | 0.914 | 0.761 | 1.151 | 0.589 |
|  |  | 0.966 | 0.953 | 0.823 | 0.500 | 0.632 | 0.977 | 1.007 | 0.818 |
|  |  | 0.837 | 1.480 | 1.197 | 1.477 | 0.953 | 1.170 | 1.161 | 0.587 |
|  | WSSV | 0.872 | 0.687 | 1.179 | 2.131 | 1.021 | 0.719 | 0.643 | 0.302 |
|  |  | 0.880 | 1.100 | 0.940 | 2.275 | 1.063 | 0.482 | 0.622 | 0.288 |
|  |  | 0.776 | 0.971 | 1.000 | 1.196 | 0.840 | 0.541 | 0.631 | 0.373 |

**Table S3.** **The data of the ratios of GSH/GSSH ralated to Figure S1B**

|  |  | 10min | 0.5h | 1h | 2h | 4h | 6h | 12h | 24h |
| --- | --- | --- | --- | --- | --- | --- | --- | --- | --- |
| Total Glutathione (μM) | PBS | 1.074 | 2.149 | 1.826 | 1.703 | 1.626 | 2.037 | 2.063 | 1.894 |
|  |  | 1.411 | 2.184 | 2.323 | 2.012 | 1.844 | 1.873 | 1.631 | 1.706 |
|  |  | 1.055 | 2.228 | 1.414 | 1.957 | 1.818 | 2.185 | 0.780 | 1.661 |
|  | WSSV | 1.841 | 0.845 | 1.462 | 1.741 | 2.477 | 1.648 | 3.071 | 2.452 |
|  |  | 1.355 | 1.357 | 1.466 | 1.871 | 2.765 | 3.391 | 2.943 | 2.862 |
|  |  | 1.421 | 1.017 | 1.562 | 1.852 | 1.540 | 3.632 | 3.444 | 2.512 |
| GSH (μM) | PBS | 0.561 | 1.220 | 0.971 | 0.971 | 0.910 | 1.128 | 1.147 | 1.115 |
|  |  | 0.728 | 1.092 | 1.204 | 1.180 | 0.930 | 1.182 | 0.955 | 0.897 |
|  |  | 0.535 | 1.119 | 0.698 | 1.149 | 0.966 | 1.270 | 0.444 | 1.006 |
|  | WSSV | 0.953 | 0.434 | 0.746 | 0.822 | 1.386 | 1.144 | 2.111 | 1.545 |
|  |  | 0.668 | 0.681 | 0.708 | 1.080 | 1.716 | 2.444 | 2.070 | 1.834 |
|  |  | 0.725 | 0.497 | 0.759 | 0.956 | 0.751 | 2.525 | 2.475 | 1.505 |
| GSSG (μM) | PBS | 0.257 | 0.464 | 0.428 | 0.366 | 0.358 | 0.455 | 0.458 | 0.389 |
|  |  | 0.342 | 0.546 | 0.559 | 0.416 | 0.457 | 0.345 | 0.338 | 0.405 |
|  |  | 0.260 | 0.554 | 0.358 | 0.404 | 0.426 | 0.458 | 0.168 | 0.327 |
|  | WSSV | 0.444 | 0.206 | 0.358 | 0.460 | 0.546 | 0.252 | 0.480 | 0.454 |
|  |  | 0.343 | 0.338 | 0.379 | 0.396 | 0.525 | 0.474 | 0.436 | 0.514 |
|  |  | 0.348 | 0.260 | 0.401 | 0.448 | 0.394 | 0.554 | 0.484 | 0.503 |
